# Integrated multi-omics analysis of oligodendroglial tumours identifies three subgroups of 1p/19q co-deleted gliomas

**DOI:** 10.1038/ncomms11263

**Published:** 2016-04-19

**Authors:** Aurélie Kamoun, Ahmed Idbaih, Caroline Dehais, Nabila Elarouci, Catherine Carpentier, Eric Letouzé, Carole Colin, Karima Mokhtari, Anne Jouvet, Emmanuelle Uro-Coste, Nadine Martin-Duverneuil, Marc Sanson, Jean-Yves Delattre, Dominique Figarella-Branger, Aurélien de Reyniès, François Ducray, POLA network, Clovis Adam, Marie Andraud, Marie-Hélène Aubriot-Lorton, Luc Bauchet, Patrick Beauchesne, Franck Bielle, Claire Blechet, Mario Campone, Antoine F. Carpentier, Ioana Carpiuc, Dominique Cazals-Hatem, Marie-Pierre Chenard, Danchristian Chiforeanu, Olivier Chinot, Elisabeth Cohen-Moyal, Philippe Colin, Phong Dam-Hieu, Christine Desenclos, Nicolas Desse, Frederic Dhermain, Marie-Danièle Diebold, Sandrine Eimer, Thierry Faillot, Mélanie Fesneau, Denys Fontaine, Stéphane Gaillard, Guillaume Gauchotte, Claude Gaultier, François Ghiringhelli, Joel Godard, Edouard Marcel Gueye, Jean Sebastien Guillamo, Selma Hamdi-Elouadhani, Jerome Honnorat, Jean Louis Kemeny, Toufik Khallil, François Labrousse, Olivier Langlois, Annie Laquerriere, Delphine Larrieu-Ciron, Emmanuelle Lechapt-Zalcman, Caroline Le Guérinel, Pierre-Marie Levillain, Hugues Loiseau, Delphine Loussouarn, Claude-Alain Maurage, Philippe Menei, Marie Janette Motsuo Fotso, Georges Noel, Fabrice Parker, Michel Peoc'h, Marc Polivka, Isabelle Quintin-Roué, Carole Ramirez, Damien Ricard, Pomone Richard, Valérie Rigau, Audrey Rousseau, Gwenaelle Runavot, Henri Sevestre, Marie Christine Tortel, Fanny Vandenbos, Elodie Vauleon, Gabriel Viennet, Chiara Villa

**Affiliations:** 1 Programme Cartes d’Identité des Tumeurs (CIT), Ligue Nationale Contre Le Cancer, 75013 Paris, France; 2 Université Pierre et Marie Curie Paris 6, Centre de Recherche de l’Institut de Cerveau et de la Moelle Epinière (CRICM), UMR 975, 75013 Paris, France; 3 INSERM U975, 75013 Paris, France; 4 CNRS, UMR 7225, 75013 Paris, France; 5 AP-HP, Groupe Hospitalier Pitié-Salpêtrière, Service de Neurologie 2-Mazarin, 75013 Paris, France; 6 Université de la Méditerranée, Aix-Marseille, Faculté de Médecine La Timone, CRO2, UMR 911, 13885 Marseille, France; 7 AP-HP, Groupe Hospitalier Pitié-Salpêtrière, Laboratoire de Neuropathologie R. Escourolle, 75013 Paris, France; 8 Département de Pathologie et Neuropathologie, Hôpital Neurologique, Hospices Civils de Lyon, 69374 Lyon, France; 9 CHU Toulouse, Hôpital de Rangueil, Service d’Anatomie et Cytologie Pathologique, 31400 Toulouse, France; 10 AP-HP, Groupe Hospitalier Pitié-Salpêtrière, Service de Neuroradiologie, 75013 Paris, France; 11 Onconeurotek, Groupe Hospitalier Pitié-Salpêtrière, 75013 Paris, France; 12 AP-HM, Hôpital de la Timone, Service d’Anatomie Pathologique et de Neuropathologie, 13885 Marseille, France; 13 Hospices Civils de Lyon, Hôpital Neurologique, Service de Neuro-Oncologie, 69374 Lyon, France; 14 Department of Cancer Cell Plasticity, Cancer Research Centre of Lyon, INSERM U1052, CNRS UMR5286, 69008 Lyon, France; 15 Université Claude Bernard Lyon 1, 69000 Lyon, France; 16 Hôpital Bicêtre, Pathology Department, 94275 Le Kremlin-Bicêtre, France; 17 CHU Saint-Pierre de la Réunion, Pathology Department, 97410 Saint-Pierre de la Réunion, France; 18 CHU Dijon, Pathology Department, 21000 Dijon, France; 19 CHU de Montpellier, Neurosurgery Department, 34925 Montpellier, France; 20 CHU Nancy, Neuro-oncology Department, 54035 Nancy, France; 21 Groupe Hospitalier Pitié-Salpêtrière, Neuropathology Department, 75013 Paris, France; 22 CHR Orléans, Pathology Department, 45000 Orléans, France; 23 Centre René Gauducheau, Medical Oncology Department, 44805 Saint-Herblain, France; 24 Hôpital Avicenne, Neurology Department, 93001 Bobigny, France; 25 Clinique des Cèdres, Medical Oncology Department, 31700 Cornebarrieu, France; 26 Hôpital Beaujon, Neurosurgery Department, 92110 Clichy, France; 27 CHU Strasbourg, Pathology Department, 67098 Strasbourg, France; 28 CHU Rennes, Pathology Department, 35033 Rennes, France; 29 Hôpital de la Timone, Assistance Publique—Hôpitaux de Marseille, Neuro-oncology Department, 13385 Marseille, France; 30 Institut Claudius Regaud, Radiotherapy Department, 31059 Toulouse, France; 31 Clinique de Courlancy, Radiotherapy Department, 51100 Reims, France; 32 Hôpital de la cavale blanche, CHU Brest, Neurosurgery Department, 29609 Brest, France; 33 Hôpital Nord, CHU Amiens, Neurosurgery Department, 80054 Amiens, France; 34 HIA Sainte-Anne, Neurosurgery Department, 83800 Toulon, France; 35 Institut Gustave Roussy, Radiotherapy Department, 94805 Villejuif, France; 36 CHU Reims, Pathology Department, 51092 Reims, France; 37 CHU de Bordeaux-GH Pellegrin, Pathology Department, 33000 Bordeaux, France; 38 Hôpital Beaujon, Neurosurgery Department, 92110 Clichy, France; 39 CHR Orléans, Radiotherapy Department, 45000 Orléans, France; 40 CHU Nice, Neurosurgery Department, 06002 Nice, France; 41 Hôpital Foch, Neurosurgery Department, 92151 Suresnes, France; 42 CHU Nancy, Pathology Department, 54035 Nancy, France; 43 CH Colmar, Neurology Department, 68024 Colmar, France; 44 Centre Georges-François Leclerc, Medical Oncology, 21079 Dijon, France; 45 Hôpital Jean Minjoz, CHU Besançon, Neurosurgery Department, 25030 Besançon, France; 46 Hôpital Dupuytren, CHU de Limoges, Neurosurgery Department, 87042 Limoges, France; 47 CHU de Caen, Neurology Department, 14033 Caen, France; 48 Hôpital Lariboisière, Neurosurgery Department, 75475 Paris, France; 49 Hospices Civils de Lyon, Hôpital Neurologique, Neuro-oncology Department, 69677 Bron, France; 50 CHU Clermont-Ferrand, Pathology Department, 63003 Clermont-Ferrand, France; 51 CHU Clermont-Ferrand, Neurosurgery Department, 63003 Clermont-Ferrand, France; 52 Hôpital Dupuytren, CHU de Limoges, Pathology Department, 87042 Limoges, France; 53 CHU Charles Nicolle, Neurosurgery Department, 76000 Rouen, France; 54 CHU Charles Nicolle, Pathology Department, 76031 Rouen, France; 55 CHU Poitiers, Neurology Department, 86000 Poitiers, France; 56 CHU de Caen, Pathology Department, 14033 Caen, France; 57 Hôpital Henri Mondor, Neurosurgery Department, 94010 Henri Mondor, France; 58 CHU Poitiers, Neurosurgery Department, 86000 Poitiers, France; 59 CHU de Bordeaux-GH Pellegrin, Neurosurgery Department, 33000 Bordeaux, France; 60 CHU Nantes, Pathology Department, 44093 Nantes, France; 61 CHU de Lille, Pathology Department, 59037 Lille, France; 62 CHU Angers, Neurosurgery Department, 49933 Angers, France; 63 Hôpital Nord, CHU Saint-étienne, Neurosurgery Department, 42270 Saint-Priest en Jarez, France; 64 Centre Paul Strauss, Radiotherapy Department, 67065 Strasbourg, France; 65 Hôpital Bicêtre, Neurosurgery Department, 94275 Le Kremlin-Bicêtre, France; 66 Hôpital Nord, CHU Saint-étienne, Pathology Department, 42270 Saint-Priest en Jarez, France; 67 Hôpital Lariboisière, Pathology Department, 75475 Paris, France; 68 Hôpital de la cavale blanche, CHU Brest, Pathology Department, 29609 Brest, France; 69 CHU de Lille, Neurosurgery Department, 59037 Lille, France; 70 HIA du Val de Grâce, Neurology Department, 75230 Paris, France; 71 Clinique des Cèdres, Pathology Department, 31023 Cornebarrieu, France; 72 CHU de Montpellier, Pathology Department, 34295 Montpellier, France; 73 CHU Angers, Pathology Department, 49933 Angers, France; 74 CHU Saint-Pierre de la Réunion, Neurology Department, 97410 Saint-Pierre de la Réunion, France; 75 Hôpital Nord, CHU Amiens, Pathology Department, 80054 Amiens, France; 76 Hôpital Beaujon, Pathology Department, 92110 Clichy, France; 77 CHU Nice, Pathology Department, 06002 Nice, France; 78 Centre Eugène Marquis, Medical Oncology, 35042 Rennes, France; 79 Hôpital Jean Minjoz, CHU Besançon, Pathology Department, 25030 Besançon, France; 80 Hôpital Foch, Pathology Department, 92151 Suresnes, France

## Abstract

Oligodendroglial tumours (OT) are a heterogeneous group of gliomas. Three molecular subgroups are currently distinguished on the basis of the *IDH* mutation and 1p/19q co-deletion. Here we present an integrated analysis of the transcriptome, genome and methylome of 156 OT. Not only does our multi-omics classification match the current classification but also reveals three subgroups within 1p/19q co-deleted tumours, associated with specific expression patterns of nervous system cell types: oligodendrocyte, oligodendrocyte precursor cell (OPC) and neuronal lineage. We confirm the validity of these three subgroups using public datasets. Importantly, the OPC-like group is associated with more aggressive clinical and molecular patterns, including *MYC* activation. We show that the *MYC* activation occurs through various alterations, including *MYC* genomic gain, *MAX* genomic loss, *MYC* hypomethylation and microRNA-34b/c down-regulation. In the lower grade glioma TCGA dataset, the OPC-like group is associated with a poorer outcome independently of histological grade. Our study reveals previously unrecognized heterogeneity among 1p/19q co-deleted tumours.

## Abstract (web summary)

Oligodendroglial tumours are characterized into three different molecular subtypes. Here, the authors use genomic data to identify a further three subgroups of 1p/19q co-deleted tumours and demonstrate an association with an aggressive phenotype.

## Graphical abstract

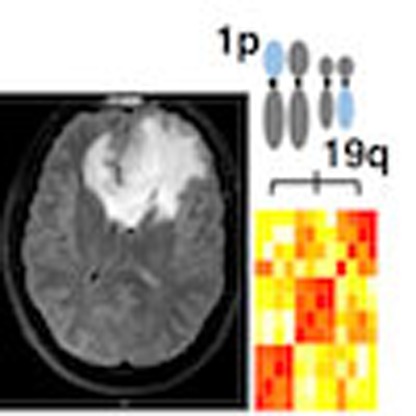

Oligodendroglial tumours (OT), that is, gliomas with an oligodendroglial differentiation, account for ∼20% of adult diffuse gliomas^1^. They form a heterogeneous group of gliomas in terms of clinical, histological and molecular profiles^2^. The survival times of OT patients range from a few years to more than 15 years. This clinical heterogeneity reflects underlying molecular heterogeneity. From a molecular point of view, three main subgroups of adults diffuse gliomas can be distinguished on the basis of two biomarkers, the 1p/19q co-deletion and the isocitrate dehydrogenase (*IDH*) mutation status^3^,^4^. Gliomas with the 1p/19q co-deletion (which are virtually all *IDH* mutated) display the best prognosis. The *IDH*-mutated gliomas, without 1p/19q co-deletion, have an intermediate prognosis. Finally, the non-1p/19q co-deleted and non-*IDH*-mutated gliomas have a poor prognosis. OT can belong to all three molecular subgroups even though pure oligodendroglial differentiation is strongly associated with the 1p/19q co-deletion^1^. Several studies have shown that this molecular classification was very robust and superior to the histological classification^5^,^6^. Accordingly, the revised World Health Organization (WHO) classification has proposed to use the *IDH* mutation and the 1p/19q co-deletion status to provide an integrated histo-molecular diagnosis of OT^7^. The aim of the present study was to assess whether the molecular classification of OT could be further refined on the basis of the integration of data from additional molecular levels.

Here we present an integrated analysis of the transcriptome, genome and methylome of 156 OT. In addition to formerly described subgroups, we report the identification of three subgroups within 1p/19q co-deleted tumours. One group is associated with more aggressive clinical and molecular patterns, including the *MYC* pathway activation. Our study reveals previously unrecognized heterogeneity among 1p/19q co-deleted tumours.

## Results

### 1p/19q co-deleted OT are molecularly heterogeneous

We used a series of 156 primary OT, 14 additional primary glioma samples and 9 normal samples referred henceforth as the Prise en charge des oligodendrogliomes anaplasiques (POLA) cohort. All samples (*n*=179) were profiled on messenger RNA (mRNA) expression arrays. MicroRNA (miRNA) sequencing was performed on 177 samples, and most of them were further profiled on single-nucleotide polymorphism (SNP) arrays (*n*=161) and DNA methylation arrays (*n*=104) as described in Supplementary Table 1.

A preliminary hierarchical consensus clustering of mRNA expression identified a subset of tumours (*n*=29), which consistently clustered with normal brain and epilepsy surgery samples. Those tumours were also assigned to the ‘cluster 0’ defined by Gravendeel *et al*.^8^ as a group of samples with a high amount of non-neoplastic brain tissue. These tumours were considered as too contaminated with normal brain tissue and therefore removed for further analyses.

**Figure 1:**
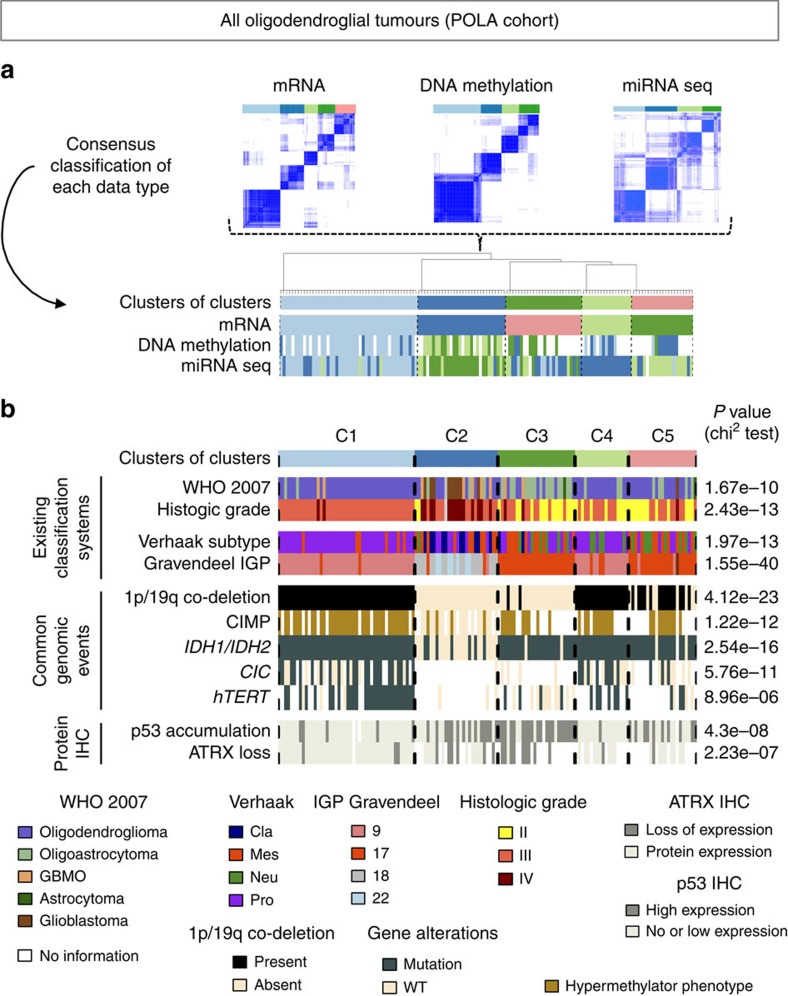
Histo-molecular characterization of the five subtypes of oligodendroglial tumours robustly identified in POLA cohort. (**a**) An integrative ‘cluster of cluster’ approach was used to define robust molecular subtypes of 141 oligodendroglial tumours. Consensus clustering was used to assign molecular classes on the basis of mRNA data, DNA methylation data and microRNA data independently. Consensus ‘clusters of clusters’ were subsequently identified on the basis of the classes labels resulting from previous independent classifications. (**b**) Clinical annotations and common genomic alterations associated to each subtype. Genomic alterations were identified through the analysis of SNP arrays. For each clinical and molecular characteristic we performed χ^2^ tests to assess the strength of association with the five-class system.

**Figure 2:**
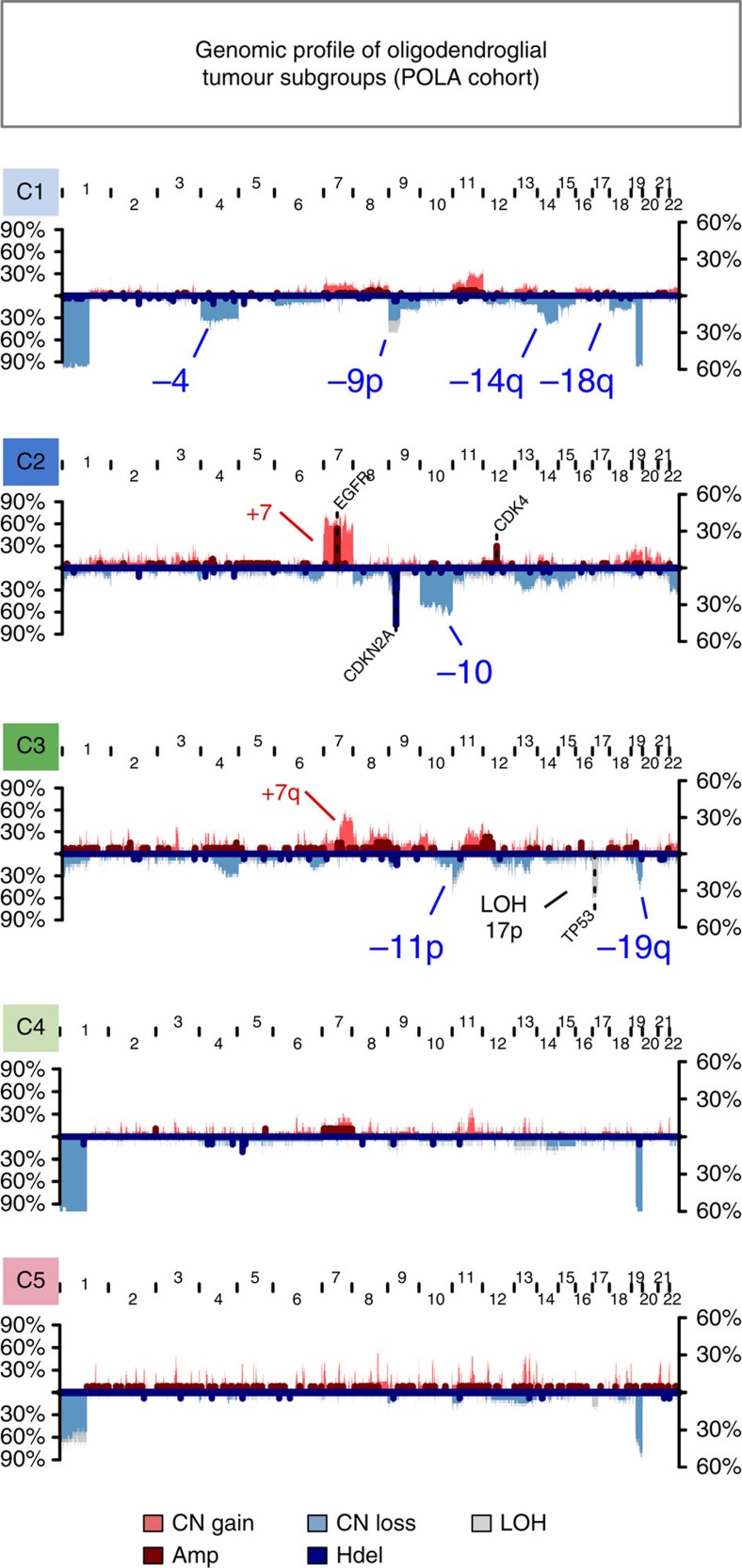
Genomic profile of oligodendroglial tumour subgroups. We analysed SNP data from 131 of the 141 oligodendroglial tumours characterized. Frequency of genomic amplifications (Amp), copy number gains (CN Gain), copy number losses (CN loss), homozygous deletions (Hdel) and copy neutral LOH events (LOH) are displayed for each of the five subtypes. Left axes show frequencies of CN gains, CN losses and LOH events. Right axes show frequencies of amplifications and homozygous deletions. The most frequently altered chromosome arms are highlightened in red (gain), blue (loss) or black (copy neutral LOH).

Unsupervised consensus clustering analysis of the 141 remaining tumour samples was then performed using three types of omics data (transcriptomic arrays (*n*=141), miRNA sequencing (*n*=137) and DNA methylation arrays (*n*=87)) independently. Transcriptome-based consensus clustering identified five robust transcriptomic subgroups, while miRNA-based and methylation-based clustering both identified four subgroups (Fig. 1a). The transcriptomic classification was highly associated with the classifications on the basis of the miRNA data (χ^2^ *P* value<1.0 × 10^−36^) and methylation data (χ^2^ *P* value <1.0 × 10^−19^). A multi-omics classification was subsequently obtained by consensus clustering of these three partitions (Supplementary Fig. 1b). Remarkably, the five resulting classes (C1–C5) nearly perfectly matched the transcriptomic classification, thereby suggesting that mRNA expression profiling would be sufficient to define robust molecular classes among OTs. We further characterized those five classes using SNP data and other histological and clinical annotations of the POLA tumours cohort. As expected the five classes were strongly associated with *IDH* mutations (χ^2^ *P* value <3.0 × 10^−16^) and with 1p/19q co-deletion status (χ^2^ *P* value <5.0 × 10^−23^) (Fig. 1b). *IDH*-mutated non-1p/19q co-deleted OT clustered into cluster C3. Their genomic profile was characterized by chromosome 7 gain (54%), chromosome 11p loss (41%) and copy neutral loss (LOH) of chromosome 17p (68%) as shown in Fig. 2. *IDH* wild-type OT formed cluster C2 and had a genomic profile as typically observed in glioblastomas, characterized by gains of chromosome 7, EGFR amplifications, CDKN2A deletions and losses of chromosome 10 (Fig. 2). As for 1p/19q co-deleted tumours, they were unexpectedly split into three different clusters C1, C4 and C5, thereby revealing previously unrecognized molecular heterogeneity among 1p/19q co-deleted OT.

### Molecular characterization of 1p/19q co-deleted OT subtypes

**Figure 3:**
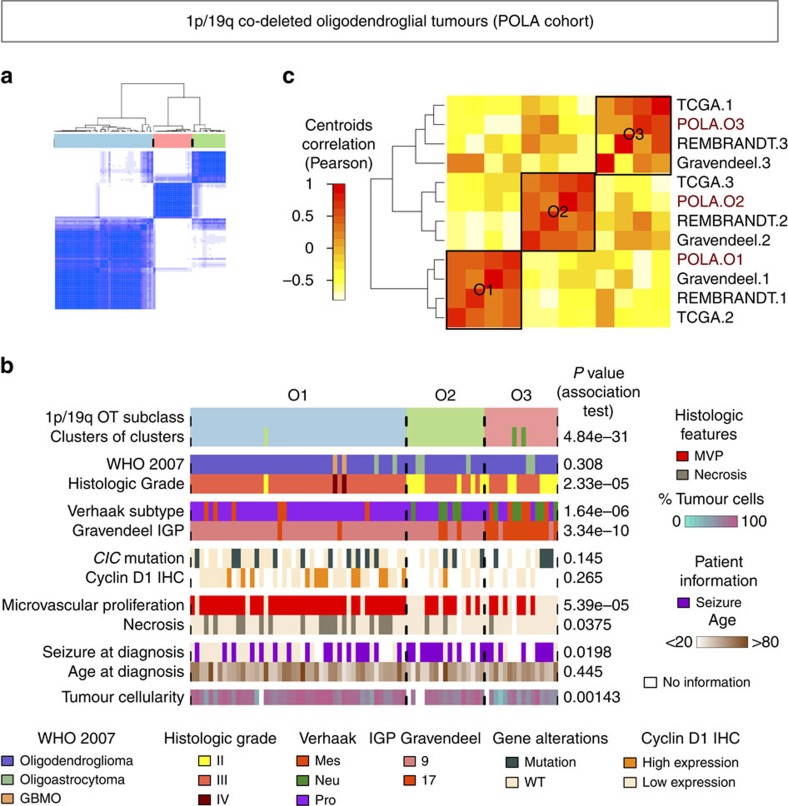
External validation of the 3 subtypes of 1p/19q co-deleted OT and further characterization. (**a**) Co-classification matrix resulting from consensus clustering analysis of mRNA data restricted to 1p/19q co-deleted OT samples (*n*=80). The strength of the blue colour is proportional to the frequency at which samples have been clustered together. (**b**) Clinical and histo-molecular annotations associated to each of the O1 (*n*=47), O2 (*n*=17), or O3 (*n*=16) subtypes of 1p/19q co-deleted tumours. We performed statistical tests for each variable to assess the strength of association with the three-class partition (Fisher tests for categorical variables and Kruskal–Wallis tests for continuous variables) and displayed the corresponding *P* values on the right. The ‘clusters of clusters’ annotation refers to the previous five-class partition presented in Fig. 1a and gives the correspondence with O1, O2 and O3 classes of 1p/19q co-deleted tumours. (**c**) Correlation matrix of class centroids derived from an unsupervised consensus classification of each dataset independently. TCGA^5^, REMBRANDT^9^ and Gravendeel^8^ co-deleted tumours could be partitioned into three stable classes on the basis of their expression data. Hierarchical clustering of the resulting class centroids (marked as 1, 2 and 3 for each dataset) on the basis of the Pearson correlation distance identifies three meta-clusters, each of them including one of the O1, O2 and O3 class centroids of our POLA discovery cohort, and one class from each other public dataset. We could therefore assign each meta-class to one of our defined O1, O2 and O3 subtypes. The three meta-classes are delimited with black squares with their corresponding ‘O’ class name.

On the basis of the previous results, we decided to focus on 1p/19q co-deleted OT. To check the robustness of the three previously related classes (C1, C4, C5), we repeated a consensus clustering analysis restricted to 1p/19q co-deleted tumours. For sake of clarity we limited this analysis to the transcriptome, which perfectly summarised multi-omics clusters (Supplementary Fig. 1). We identified three robust subgroups O1, O2, O3 matching almost perfectly the previously identified clusters C1, C4 and C5, respectively (χ^2^ *P* value=1.0 × 10^−30^, Fig. 3a,b). To validate the three subgroups of 1p/19q co-deleted OT, we performed an unsupervised consensus clustering of mRNA data using the 1p/19q co-deleted OT from three additional public cohorts (The Cancer Genome Atlas^5^ (TCGA), Gravendeel *et al*.^8^, and REMBRANDT project^9^, Supplementary Fig. 2). As in our dataset, unsupervised consensus clustering optimally partitioned each public dataset into three clusters. We analysed the correlation patterns of class centroids in both our dataset and public datasets, and observed a high similarity between all three-group partitions (Fig. 3c), thereby confirming our findings.

In the POLA cohort, patients in O1 tended to be older than patients in O2 and O3 (48.7 years vs 44.8 years, *t*-test *P* value=0.08) and had less frequently seizures at diagnosis (43 vs 79%, Fisher test *P* value=0.001). This may be related to the fact that grade II oligodendrogliomas were mostly present in O2 and O3, while O1 tumours consisted nearly exclusively of anaplastic oligodendrogliomas (Fisher test *P* value=9.9 × 10^−6^). Consistently, O1 was significantly enriched in tumours demonstrating microvascular proliferation (89 vs 45%, Fisher test *P* value=2.0 × 10^−5^) and necrosis (36 vs 10%, Fisher test *P* value=0.001). The two 1p/19q co-deleted tumours classified as glioblastomas with oligodendroglioma component (GBMO) according to the 2007 WHO classification clustered with O1. The genomic profile of O1 tumours differed from O2 and O3 tumours with significantly higher frequencies of chromosomes 4, 9p, 14q and 18q losses, even when considering only grade III tumours (see Fisher tests *P* values in Supplementary Fig. 3); 66% of O1 tumours showed at least 1 loss of those 4 chromosomal regions, and 32% of O1 tumours had at least 2 or more regions lost. Tumour cellularity was higher in O1 and O2 tumours than in O3 tumours suggesting that this last subtype may have a more infiltrative growth pattern.

According to Gravendeel’s and Verhaak’s classifications^8^,^10^, most tumours within cluster O1 and O2 were classified as IGP 9 and as proneural, while most tumours within O3 were classified as IGP17 and as either proneural, neural or mesenchymal.

**Figure 4:**
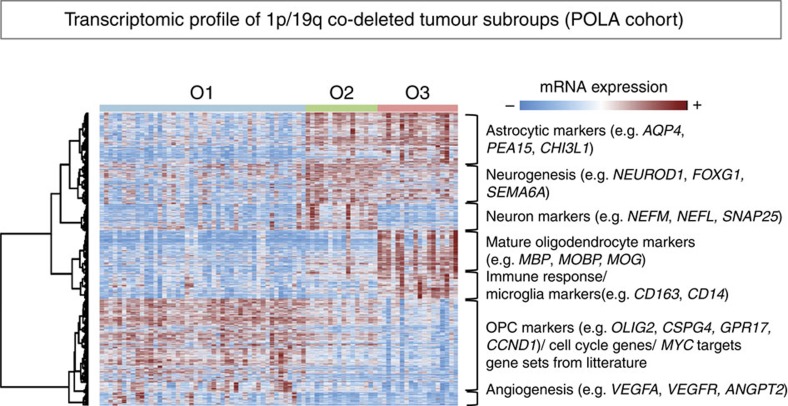
Heatmap of mRNA expression profile from the top most up-regulated probe sets in each subtype. We performed moderated *t*-tests to analyse differential expression of Affymetrix HG-U133 Plus 2.0 arrays probe sets in each subtype compared to the others, and selected the top 1,000 probe sets with the highest fold-change among all significantly deregulated probe sets (*P* value<0.05, no adjustments for multiple comparisons). For each of the eight clusters of probe sets highlighted on the heatmap we performed gene-set enrichment analysis and annotated the clusters on the right with the most relevant significantly enriched gene sets (hypergeometric test *P* value<0.05) and corresponding relevant gene markers.

Tests for differential gene expression between subgroups and gene-set enrichment analysis demonstrated that the three subgroups were characterized by the expression of specific markers of differentiation (Fig. 4; Supplementary Table 2; Supplementary Data 1). O1 tumours were characterized by a higher expression of oligodendrocyte precursor cell (OPC) markers, especially *GPR17* (ref. 11) and *CCND1* (ref. 12) which was validated by immunohistochemistry (Fig. 3b); O2 tumours strongly overexpressed neuron markers^13^,^14^ and genes implicated in neurogenesis; and O3 tumours specifically expressed mature oligodendrocyte markers^14^,^15^. Astrocytic markers were overexpressed in both O2 and O3 compared to O1.

O1 tumours also overexpressed cell-cycle genes, genes implicated in glioma angiogenesis, and key epithelial–mesenchymal transition markers (for example, *TWIST1*, *SNAI2* and *POSTN*), a feature associated with tumour progression in gliomas^16^ and observed in glioblastomas^17^. The most striking differential activity among the oncogenic pathways was observed in O1 tumours where several gene sets reflecting *MYC* activity were found among the most significantly deregulated gene sets (GSA score>1, *P* value<0.05; Supplementary Table 2).

As in our dataset, O1 TCGA tumours tended to originate from older patients (mean age at diagnosis 51.8 vs 42.8 years, *t*-test *P* value=0.08). They were also associated to a higher grade and with more frequent losses of chromosomal arms 9p and 14q (Fisher test *P* values=0.04 and 0.009, respectively, Supplementary Fig. 4a). With the exception of *NOTCH1*, which was almost never found mutated in O3 subtype, TCGA exome data analysis did not identify any mutation significantly associated with a specific subgroup (Supplementary Fig. 4b). As in the POLA dataset, *CIC* mutations were found in all subgroups. Gene enrichment analysis in each TCGA class was consistent with O1, O2 and O3 gene expression characteristics in the POLA cohort (Supplementary Fig. 5). In particular, a striking enrichment in gene sets related to the *MYC* pathway was also observed in TCGA O1 tumours.

### Multi-level deregulation of *MYC* activity in O1 tumours

The *MYC* pathway activity was assessed in each tumour as the mean expression of *MYC* target genes. Consistently this measure was higher in O1 than in O2 and O3 tumours (*t*-test *P* value <1.0 × 10^−3^; Supplementary Fig. 6). *MYC* expression profile was also higher in O1 tumours (*t*-test *P* value ≤0.013; Supplementary Fig. 6). To determine which molecular mechanisms could trigger *MYC* activation in O1 tumours, we looked for genomic, epigenetic and post-transcriptional events reported to enhance the *MYC* pathway activity in both the POLA and TCGA datasets.

**Figure 5:**
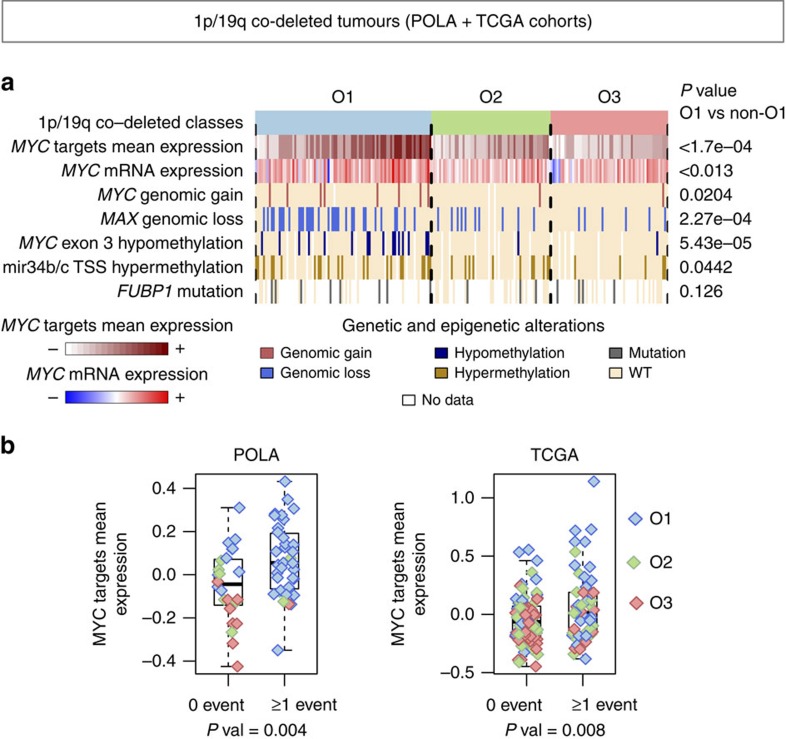
Overview of *MYC*-related genomic and epigenomic alterations frequently observed in O1 tumours. (**a**) Summary of *MYC*-related genetic and epigenetic alterations in pooled data from both POLA (80 co-deleted gliomas) and TCGA (131 co-deleted gliomas) cohorts. The pooled sample sizes of O1, O2 and O3 classes are respectively 90, 61 and 60 tumours. The mean expression of *MYC* targets was computed for each sample to get a measure of *MYC* activation. (**b**) Relation between the presence of at least one *MYC* deregulation event (*MYC* genomic gain, *MAX* genomic loss, *MYC* exon 3 hypomethylation, mir34b/c TSS hypermethylation) and *MYC* activity measured through the mean expression of *MYC* targets. For each dataset, *y* axis show the mean expression values after centring on the samples. For each box and whiskers plot, bottom and top of the boxes are the first and third quartile of the data and whiskers represent the lowest (respectively highest) data point still within 1.5 interquartile range of the lower (respectively upper) quartile. Bold lines represent median values.

At the genomic level, gains of *MYC* locus and losses of *MAX* (Myc-associated factor X), a negative regulator of *MYC*^18^, were more frequent in O1 than in O2 and O3 tumours (*t*-test *P* values=0.02 and 0.0002, respectively; Fig. 5a). At the epigenetic level, *MYC* exon 3 hypomethylation^19^,^20^ was specifically associated with O1 tumours in both datasets (*P* value<0.0001) and correlated with a higher *MYC* expression (Fig. 5a and Supplementary Fig. 7). In addition, two negative regulators of *MYC*, mir34b and mir34c^21^,^22^,^23^, were down-regulated in O1 tumours and their transcription start sites, lying within the mir34b/c CpG island, were hypermethylated in both POLA and TCGA datasets (Supplementary Fig. 7).

**Figure 6:**
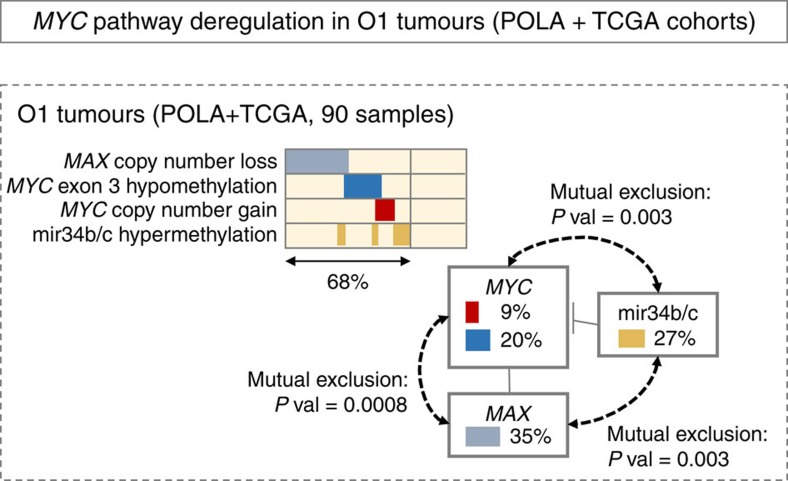
Focus on O1 tumours and their specific *MYC* signalling related alterations. Percentages refer to the proportion of O1 tumours (pooled data from POLA and TCGA) harbouring the alterations. *P* values refer to one-sided binomial tests, which assess the probability that two of the three genetic loci are both altered in the same tumour sample.

These four mechanisms—*MYC* genomic gain (9% of O1 tumours), *MAX* genomic loss (35% of O1 tumours), *MYC* exon 3 hypomethylation (20% of O1 tumours) and mir34b/c locus hypermethylation (28% of O1 tumours)—were not all required to observe an increase of *MYC* activity. Consistently, *MYC* targets mean expression increased in samples having at least one of this events, in both POLA and TCGA datasets (Fig. 5b). Moreover, *MYC* alterations (genomic gain or exon 3 hypomethylation) tended to be exclusive with mir34b/c locus hypermethylation (binomial test *P* value=0.003; Fig. 6) and *MAX* genomic losses (binomial test *P* value=8.0 × 10^−4^; Fig. 6). In the TCGA dataset, *MYC* gain and *MYC* exon 3 hypomethylation never occurred with the *FUBP1* mutation, which is thought to increase *MYC* activity^24^.

Taken together, these results suggest that various molecular mechanisms concur to *MYC* activity in O1 tumours: genomic alterations, hypomethylation and down-regulation of its silencers mir34b and mir34c through hypermethylation of their promoter region.

### Association with survival

**Figure 7:**
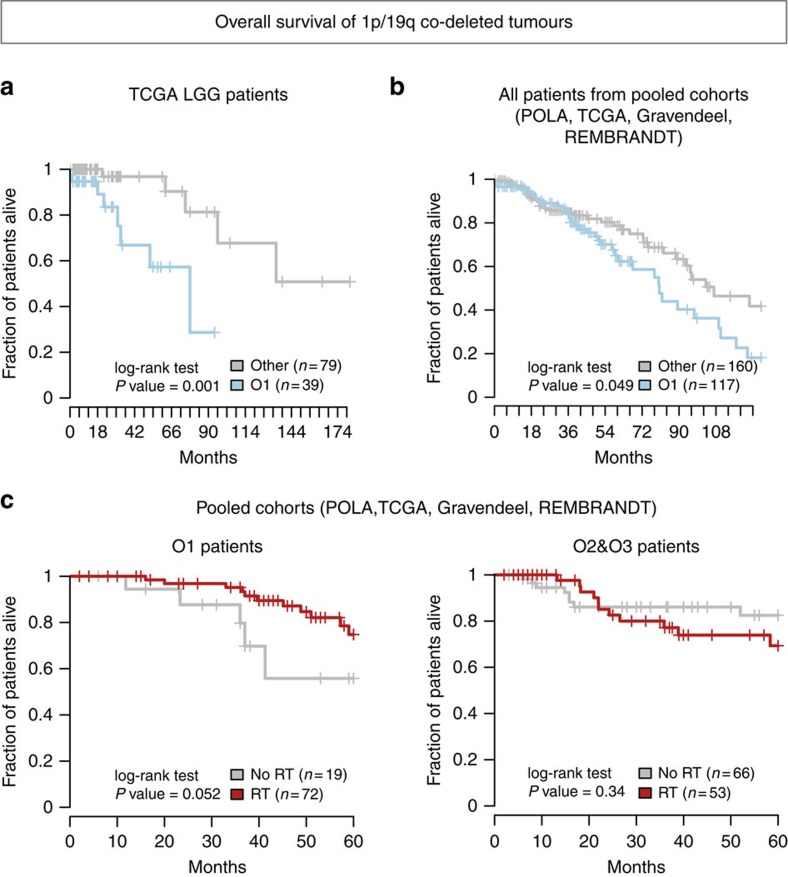
Overall survival of 1p/19q co-deleted tumours (**a**) Overall survival of TCGA patients with 1p/19q co-deleted tumours according to O1 subtype membership. We used the available clinical data from 118 patients with co-deleted tumours (59 with grade III tumours and 59 with grade II tumours). (**b**) Overall survival of all 278 patients with 1p/19q co-deleted tumours after pooling patients with available clinical data from TCGA (*n*=118 patients), POLA (*n*=80 patients), Gravendeel (*n*=42 patients) and REMBRANDT (*n*=37 patients) cohorts. (**c**) Overall survival of O1, and other O2 or O3 patients who did or did not receive radiotherapy as initial treatment after surgery. We used data from 222 patients with 1p/19q co-deleted tumours that were pooled from the four cohorts: POLA (*n*=75 patients), TCGA (*n*=66 patients), REMBRANDT (*n*=49 patients) and Gravendeel (*n*=32 patients). Patients were included if their treatment and survival data were available and if they had not deceased within the first 3 months after diagnosis so that they could have effectively received radiotherapy. For each subgroup of patients (with O1, O2 or O3 tumours) we compared the five-year survival of patients treated with an initial radiotherapy (RT, red curves)—combined or not with chemotherapy—against patients who had not received initial radiotherapy and were managed with initial follow-up or with chemotherapy alone (No RT, grey curves).

In the POLA, Gravendeel and REMBRANDT cohorts we did not observe any significant association between O1/O2/O3 partition and prognosis. However, due to still limited follow-up, median overall survival was not reached in the POLA cohort. As for Gravendeel and REMBRANDT cohorts, their sizes were limited and their median survival (6 years) was not fully representative of the median survival usually observed in 1p/19q co-deleted tumours^25^ (>10 years) (Supplementary Table 3). In contrast, a remarkable association with prognosis was observed within the TCGA cohort (Fig. 7a), and was independent of grade and age (Supplementary Fig. 8). Consistently, 1p/19q co-deleted tumours with the highest *MYC* activity score had a worse prognosis (log-rank *P* value=0.01; Supplementary Fig. 9). When pooling survival data from the four cohorts, there was a trend towards an association of O1 subtype with a worse prognosis (log-rank *P* value=0.049; Fig. 7b). Moreover, also consistent with the higher aggressiveness of O1 subtype, analysis of all patients for whom treatment data was available showed that an initial treatment without radiotherapy (that is, with initial follow-up or with chemotherapy alone) was associated with shorter survival in O1 but not in O2 and O3 tumours (log-rank *P* value=0.052; Fig. 7c).

## Discussion

In agreement with the TCGA low-grade glioma study, we show here a strong correlation between the classification of OT based either on the different omics separately or on the integrated clustering of the omics (‘cluster of clusters’ analysis) and the 1p/19q co-deletion, and *IDH* mutation status^5^. These findings further illustrate the robustness of the subgroups defined by these two biomarkers and support their integration into the revised classification of diffuse gliomas^6^. Moreover, because of its enrichment in 1p/19q co-deleted gliomas, our study identified three expression-based subgroups within these tumours and robustly reproduced this classification in public datasets through unsupervised analysis of 1p/19q co-deleted glioma samples.

The three subgroups of 1p/19q co-deleted tumours had different patterns of differentiation related to OPC, astrocytic, neuronal and oligodendrocytic marker genes expression. O1 OPC-like gliomas had a more aggressive histological and genomic profile with frequent chromosome 9p and 14q losses. It remains to be determined whether these three subgroups correspond to different oncogenic pathways or to different steps during oligodendrogliomagenesis. Yet, the absence of clear differences regarding the somatic mutational landscape of the three subgroups rather discards the first hypothesis. The second hypothesis is supported by the higher age observed among O1 patients in both POLA and TCGA cohorts and by the fact that *MYC* activation which was frequently observed in O1 tumours, has recently been implicated in the malignant progression of *IDH* mutant gliomas^26^. Besides, strong evidence suggests that OPC are the cell of origin of oligodendrogliomas^27^. These cells can differentiate into oligodendrocytes, astrocytes and may also differentiate into neural cells^28^. Therefore, in the more differentiated O2 and O3 subgroups, tumour cells could still be able to differentiate, while this differentiation capacity would be lost in the O1 OPC-like tumours as additional genomic alterations are acquired. The study of the gene expression profile of initial and recurrent tumours would be of great interest to determine whether tumours from the differentiated groups can evolve into OPC-like tumours over time. The better prognosis of the differentiated subgroups also argue towards the use of differentiation therapies in O1 tumours, such as inhibitors of the membrane receptor *GPR17*, which was highly expressed in the OPC-like group and has been suggested to block OPC differentiation^29^,^30^. Interestingly, such inhibitors are being developed to promote myelin repair in multiple sclerosis^29^. In the O1 group, the *MYC* pathway appeared as a particularly important oncogenic pathway. *FUBP1* inactivating mutations are thought to activate *MYC*^24^. However, they were not significantly associated with the OPC-like group (Supplementary Fig. 4). Here, we identified four distinct molecular mechanisms that could concur to increase *MYC* activity in the OPC-like group: *MYC* locus genomic gain, *MAX* locus genomic loss, hypomethylation of *MYC* exon 3, and down-regulation of *MYC* silencers mir34b and mir34c through promoter hypermethylation. *MYC* locus genomic gain, together with *MAX* and *FBXW7* locus genomic losses have been suggested to activate the *MYC* pathway during the malignant progression of *IDH*-mutated gliomas^26^. *FBXW7* locus genomic loss was not significantly associated with the OPC-like group, but *MAX* locus genomic loss at 14q was observed in 35% of O1 tumours. *MAX*, a *MYC*-associated factor, is a tumour suppressor gene whose mutations cause hereditary pheochromocytoma^31^. *MYC* locus genomic gain at 8q24 was observed in about 10% of O1 tumours. Interestingly, a strong association has been shown between 1p/19q co-deleted *IDH*-mutated gliomas and SNPs mapping to the 8q24 locus, which is rich in long non-coding RNA that may modulate *MYC* expression^32^. Hypomethylation of *MYC* exon 3 at the same CpG position than in our O1 tumours (Chr8: 128,752,988-hg19, GRCh37) has been reported in myeloma, leukaemia, B-cell malignancies and colorectal cancer^19^,^20^,^33^. This particular site seems to be important for *MYC* expression auto-regulation. In colorectal carcinoma, partial hypomethylation of this position was associated with the deregulation of cell proliferation. Mir34b and mir34c have been broadly reported to be negative regulators of *MYC*, and silencing of miR34b/c locus through promoter hypermethylation has been reported to up-regulate *MYC* expression^21^,^22^,^23^. However, 32% of O1 tumours didn’t harbour any of these four alterations despite showing a high *MYC* activity. Further analysis may identify other alterations impacting the *MYC* signalling pathway. OPC-like tumours might be candidates for strategies aiming at inhibiting *MYC* activity such as bromodomain and extraterminal bromodomain (BET) inhibition that has been shown to suppress *MYC* transcriptional activity in several cancers^34^ and to inhibit cell growth in IDH1-mutant glioma primary cell cultures^26^.

The clinical significance of the three gene expression subgroups of 1p/19q co-deleted tumours remains to be determined since association with survival was only observed in the TCGA dataset and not in the three other cohorts. However, the poorer outcome associated with classification into the O1 group and with high *MYC* activity would be in line with previous studies showing that (1) necrosis, 9p loss and a high number of genomic alterations are associated with worse prognosis in 1p/19q co-deleted tumours^35^,^36^, and (2) increased *MYC* activity is associated with malignant progression and worse prognosis in *IDH*-mutated tumours^26^,^37^. Identifying patients with 1p/19q co-deleted tumours with poorer outcome is an important issue. Since these tumours are usually chemo sensitive, these patients might be candidates to receive more intensive chemotherapy regimens. On the other hand, patients with a favourable molecular profile might be the best candidates to benefit from less intensive treatment strategies, for example, initial treatment with chemotherapy alone to reduce the potential side effects of brain radiotherapy. The present study suggests that such a strategy might be appropriate in O2 and O3 but not in O1 tumours. Future studies will have to determine efficient molecular markers to rapidly label 1p/19q co-deleted patients according to this stratification.

## Methods

### Patient samples and consent

Samples were obtained with informed and written consent after approval of the institutional review boards of respective hospitals participating in the POLA network. All patients were aged 18 years or older at diagnosis, and tumour histology was centrally reviewed and validated according to WHO guidelines^38^. A total of 179 samples were included in this study: 156 gliomas with an oligodendroglial phenotype, as well as 11 glioblastomas, 2 diffuse astrocytomas, 9 normal brain samples and 1 NOS sample. A summary of each sample of the tumour cohort and respective pathological information on the patients is provided in Supplementary Table 1.

### DNA and RNA extraction

DNA and total RNA were extracted from frozen tumour samples using the iPrepChargeSwitch Forensic Kit and the RNeasy Lipid Tissue Mini Kit (Qiagen), respectively. DNA and RNA integrity and quantity were assessed on the basis of the quality control criteria established by CIT (Cartes d’Identité des Tumeurs) programme protocols (http://cit.ligue-cancer.net). A 1-μg volume from each DNA and RNA sample was used for SNP array experiments (outsourced to the Integragen Company Paris, France) and to perform the gene expression analysis, respectively.

### SNP arrays analysis

Illumina SNP arrays were used to analyse the DNA samples from 161 tumour samples (74 Illumina HumanCNV610-Quad v1.0, 52 HumanCNV370, 34 HumanOmniExpress-12v1 and 1 HumanCore-12v1). Integragen SA (Evry, France) carried out hybridization, according to the manufacturer’s recommendations. The BeadStudio software (Illumina) was used to normalize raw fluorescent signals and to obtain log R ratio (LRR) and B allele frequency (BAF) values. Asymmetry in BAF signals due to bias between the two dyes used in Illumina assays was corrected using the tQN normalization procedure.^39^ We used the circular binary segmentation algorithm^40^ to segment genomic profiles and assign corresponding smoothed values of log R ratio and B allele frequency. The Genome Alteration Print method was used to determine the ploidy of each sample, the level of contamination with normal cells and the allele-specific copy number of each segment^41^.

### mRNA expression profiling and analysis

The IGBMC Microarray and Sequencing Platform performed mRNA expression profiling using HumanGeneChip HG-U133 Plus 2.0 arrays (Affymetrix) for the 179 samples from the study. We used the RMA algorithm (Bioconductor affy package) to normalize the data. Probe set intensities were then averaged per gene symbol.

We used the Bioconductor ConsensusClusterPlus package for consensus clustering analysis and identification of homogeneous gene expression clusters. The 5% most variant probe sets were selected to determine the consensus partitions of the data set in K clusters (for *K*=2, 3, ..., 8). Computations were performed on the basis of the 1,000 resampling iterations of hierarchical clustering, using Pearson’s dissimilarity as the distance metric and Ward’s method for linkage analysis. To determine the optimal number of clusters, we used the cumulative distribution functions (CDFs) of the consensus matrices and considered both the shape of the functions and the area under the CDF curves, as previously described^42^.

We used the Bioconductor package limma to test for gene differential expression between different conditions^43^.

### DNA methylation profiling and analysis

We analysed whole-genome DNA methylation in 104 tumour samples using the Illumina Infinium HumanMethylation450 Beadchips. Integragen SA (Evry, France) carried out microarray experiments and hybridized to the BeadChip arrays following the manufacturer’s instructions. Illumina GenomeStudio software was used to extract the beta value DNA methylation scores for each locus together with detection *P* values.

As described elsewhere^44^, we replaced data points with detection *P* value>0.05 with ‘NA’ values. We also masked data points as ‘NA’ for probes that contained SNPs or overlapped with a repetitive element that was not uniquely aligned to the human genome or regions of insertions and deletions in the human genome. Homogeneous tumour subgroups with similar methylation profiles were identified using consensus clustering. We used the Bioconductor package ConsensusClusterPlus as described above, using the 5% most variant CpG sites, the Euclidean distance metric, and R “ward.D” method for linkage analysis.

We determined CpG Island Methylator Phenotype (CIMP) by restricting the consensus clustering analysis to CpG sites located within CpG islands. Samples with a CIMP phenotype were determined according to the classification results from the partition in two classes. Samples falling within the class showing strong hypermethylation were assigned a positive CIMP status.

### miRNA profiling and analysis

miRNA profiling was performed on 177 samples. A PCR barcoding method^45^ was used to prepare multiplexed miRNA libraries that were sequenced by Integragen SA (Evry, France) on an Illumina HiSeq 2000 sequencer. Image analysis, base calling, demultiplexing and conversion of BCL to FASTQ format were performed using Illumina CASAVA 1.8.2 software. MirExpress software^46^ was used to remove adaptor sequences. MiRanalyzer0.3 software^47^ was used to process FASTA files for each sample and to quantify read counts for each miRNA referenced in mirBase74 v18.

Unsupervised classification was performed using 757 miRNAs that were expressed (>10 reads) in at least two samples. The miRNA counts were log2 transformed, divided by the total number of reads in each sample and centred on the mean expression level of each gene. Consensus clustering was performed as described above. Pearson’s dissimilarity was used as the distance metric and Ward’s method for linkage analysis. We determined the optimal number of clusters on the basis of the CDF curves.

We used the Bioconductor package limma to test for microRNA differential expression between different conditions^43^.

### Immunohistochemical staining

Immunohistochemistry was performed on 4-μm-thick sections of formalin-fixed paraffin embedded blocks with a ventana Benchmark XT Device. The following antibodies were used after antigen retrieval to assess ATRX (anti-ATRX, Sigma, polyclonal, dilution 1/400), p53 (anti-p53, Dako clone DO.7, dilution 1/200) and CCND1 (anti-CCND1, Ventana, clone SP4). p53 protein was defined as ‘highly expressed’ when we observed a strong nuclear expression in more than 10% of the nuclei.

### *IDH* and *CIC* mutations

*IDH1* codon 132 and *IDH2* codon 172 were sequenced using the Sanger method with the following primers: IDH1-Forward: TGTGTTGAGATGGACGCCTATTTG; IDH1-Reverse: TGCCACCAACGACCAAGTC; IDH2-Forward: GCCCGGTCTGCCACAAAGTC and IDH2-Reverse: TTGGCAGACTCCAGAGCCCA.

Coding exons (1–20) of the *CIC* gene were first amplified using primers used by Gleize *et al*.^48^. Primers are available in Supplementary Table 4. PCR products were purified conforming to the Agencourt AMPure XP PCR purification protocol (Beckman Coulter) with the Biomek 3000 Automation Workstation. Universal tailed amplicon resequencing approach (454 Sequencing Technology, Roche) was used for the sequencing of coding exons of CIC. Sequences analysis was performed using CLC Genomics Workbench software.

### pTERT mutations

The promoter region of *TERT* gene was amplified as follow: TERT-F: GGCCGATTCGACCTCTCT and TERT-R AGCACCTCGCGGTAGTGG ; 3 min at 94 °C; 35 cycles at 94 °C—15 s, 60 °C—45 s, 72 °C—1 min, with a final step at 72 °C for 8 min. PCR products were then purified with the Agencourt AMPure XP PCR purification protocol (Beckman Coulter). Purified PCR products were used as templates for the sequencing reaction performed with the Big-Dye Terminator Cycle Sequencing Ready Reaction (Perkin Elmer). Extension products were purified with the Agencourt CleanSEQ protocol according to manufacturer’s instructions (Beckman Coulter). Purified sequences were analysed on an ABI Prism 3730 DNA Analyzer (Applied Biosystems).

### Cluster of clusters analysis

We performed a consensus clustering of 85 and 141 tumours samples on the basis of the results from mRNA, DNA methylation and miRNA consensus clustering analyses. The samples were clustered on binary variables for each of the previously defined classes: five variables for mRNA classes, four variables for DNA methylation classes and four variables for miRNA classes. For each sample, the class variables had values 1 when the sample was in the class, 0 if in another class of the partition, ‘NA’ when the sample was not classified at the given molecular level. Pearson’s dissimilarity was used as the distance metric and Ward’s method was used for linkage analysis.

### Analysis of public data

We downloaded TCGA Low Grade Glioma data with last update on 17th October 2014. 1p/19q co-deleted status was assigned by using Gistic2 results by chromosome arm as found on the TCGA data portal. A total of 131 gliomas were labelled as 1p/19q co-deleted.

REMBRANDT and Gravendeel mRNA data were downloaded from public databases (accession codes are respectively GSE16011 and E-MTAB-3073). For both datasets, 1p/19q co-deleted status was assigned using mRNA expression data. For each sample we computed the centred mean expression values of probe sets located on chromosome arms 1p and 19q and optimized the two-class partition (1p/19q co-deleted vs non-1p/19q co-deleted) of the samples according to these two values. We labelled respectively 42 and 58 gliomas as 1p/19q co-deleted in Gravendeel and REMBRANDT cohorts.

REMBRANDT survival data was downloaded from NIH (http://rembrandt.nci.nih.gov) in august 2014 and treatment information from the G-DOC plus portal (http://gdoc.georgetown.edu/gdoc/).

### Validation of classification results on public datasets

We validated our mRNA classification results by applying the same unsupervised classification approach on the 1p/19q co-deleted samples of three additional public sample cohorts (TCGA Low Grade Glioma, Gravendeel cohort and REMBRANDT cohort). Then, for each class of each dataset, we computed a centroid profile on the basis of the samples within the class as the mean expression of the 10% most variant genes within the dataset. For each pair of classes to be compared, the 10% most variant genes were selected among the genes which were measured in both datasets. We could then compare our initial classification system to the ones achieved on each public dataset using pairwise correlations between centroids to measure the inter-dataset similarity of the classes.

### Gene-set enrichment analysis

We used the R package GSA^49^ to perform gene-set enrichment analysis for each molecular subtype compared to the others. Gene-set members lists were retrieved online from MSigDB, GO and SMD databases. Additional gene lists were added to this main set on the basis of specific publications of interest: OPC markers from Dougherty *et al*.^15^, *VEGF* activity markers from Dieterich *et al*.^50^ and *MYC* targets from Zeller *et al*.^51^. Gene list from Zeller *et al*. was also used to assign to each tumour a score of *MYC* activation on the basis of the mean expression of its targets.

### Alterations in the *MYC* pathway

Genomic gains of *MYC* and genomic losses of *MAX* were estimated from the gain normal loss (GNL) values computed from SNP arrays. In POLA dataset, tumours verifying GNL=1 (resp. GNL=−1) for all SNP positions within *MYC* (resp. *MAX*) genomic region were considered to have a genomic gain of *MYC*. For TCGA dataset we used the public GNL data which are given at gene level only: Tumours with GNL=1 (resp. GNL=−1) for *MYC* (resp. *MAX*) were assigned a positive status for *MYC* (resp. *MAX*) genomic gain.

Hypomethylation of *MYC* exon 3 was measured from DNA methylation arrays. For both POLA and TCGA datasets we considered that tumours were relatively hypomethylated on *MYC* exon 3 if the beta value at CpG position cg00163372 was <0.5.

Hypermethylation of mir34b/c genomic locus was also measured from DNA methylation arrays. We identified four CpG positions within CpG island on mir34b/C locus promoter region that were hypermethylated in O1 tumours (cg22879515, cg21881253, cg13767940 and cg23211240), and used the cg22879515 position to define mir34b/c hypermethylation in both POLA and TCGA tumours. For each dataset we defined a tumour as hypermethylated for the locus if the beta value was greater than the mean beta value plus twice the standard deviation.

## Additional information

**Accession codes:** The mRNA expression data, DNA methylation data and miRNA sequencing data have been deposited in ArrayExpress database under accession codes E-MTAB-3892, E-MTAB-3903 and E-MTAB-3901, respectively. The SNP array data has been deposited in the ArrayExpress database under accession codes E-MTAB-3905(Illumina Human610 Quad), E-MTAB-3907(Illumina HumanCNV370), E-MTAB-3896(Illumina HumanCore) and E-MTAB-3902(Illumina HumanOmniExpress).

**How to cite this article:** Kamoun, A. *et al*. Integrated multi-omics analysis of oligodendroglial tumours identifies three subgroups of 1p/19q co-deleted gliomas. *Nat. Commun.* 7:11263 doi: 10.1038/ncomms11263 (2016).

## Supplementary Material

Supplementary Information Supplementary Figures 1-9 and Supplementary Tables 1-4

Supplementary Data 1 Top most up-regulated probe sets in each subtype. We performed moderate t-tests to analyse differential expression of probe sets in each subtype compared to the others and selected the top 100 probe sets with the highest fold-change among all significantly deregulated probe sets (p-value < 0.05) for each class.
