# Supplementary material for: Integrated multi-omics analysis of oligodendroglial tumours identifies three subgroups of 1p/19q co-deleted gliomas
Source: Nat Commun. 2016 Apr 19;7:11263. doi: 10.1038/ncomms11263 (PMC4838899; doi:10.1038/ncomms11263)
Supplement: Supplementary Information — Supplementary Figures 1-9 and Supplementary Tables 1-4 [file ncomms11263-s1.pdf]

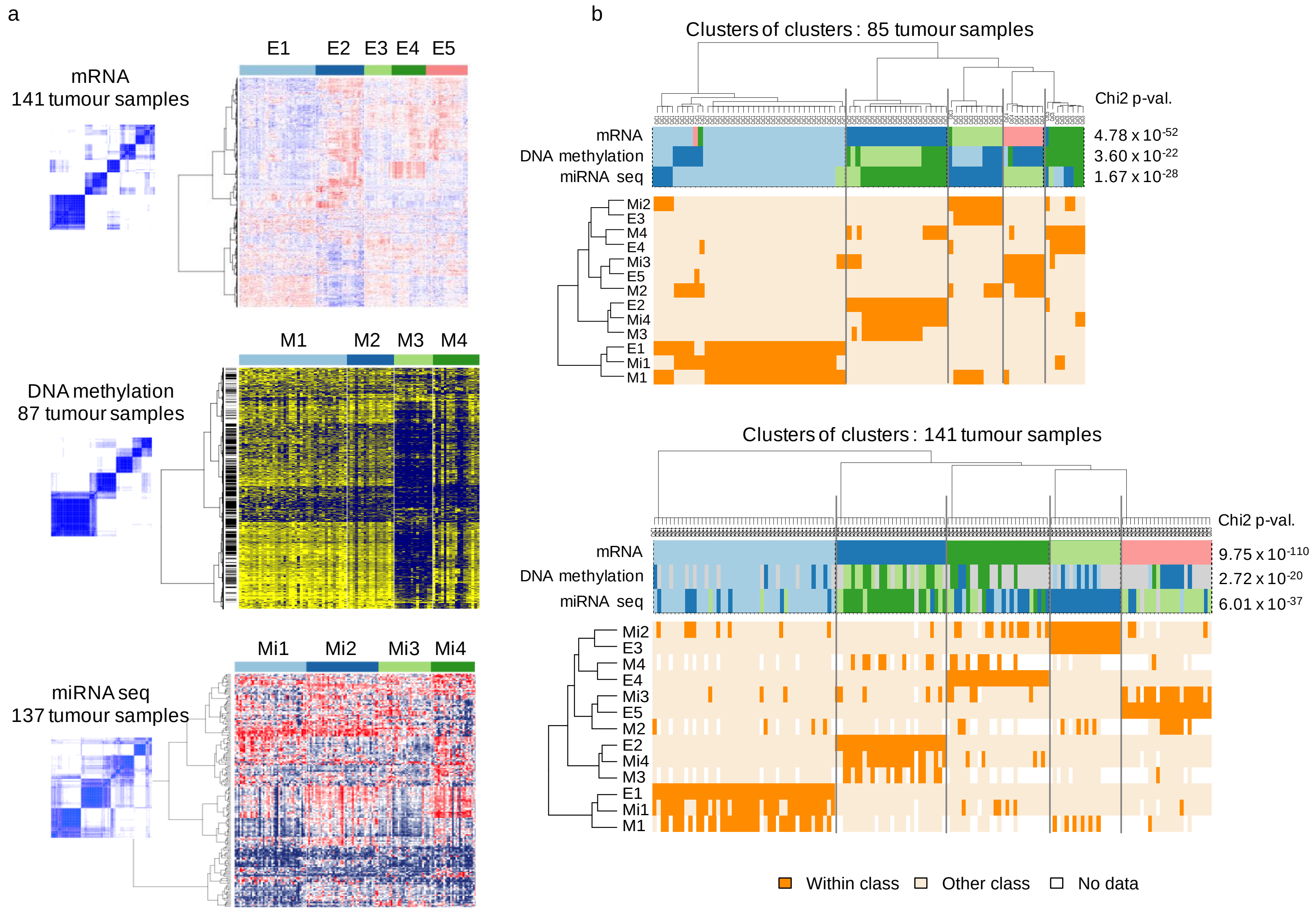

Supplementary Figure 1: « Cluster of cluster » analysis of 141 tumours. (a) Independent clustering consensus results using mRNA expression, DNA methylation, and miRNA expression data. For each data type, co-classification matrices for the optimal number of classes are on the left, and heatmaps of the most variant data ordered by sample class are on the right. **(b)** Consensus clustering results based on classes' membership resulting from the clustering analysis of each data type independently, either considering samples that were profiled by each of the 3 technology (85 tumour samples), or all the samples that were profiled at least by one technique (141 tumour samples). Samples are ordered according to the resulting clustering and the p-values refer to the association of this clustering with each of the independent clustering displayed on panel (a).

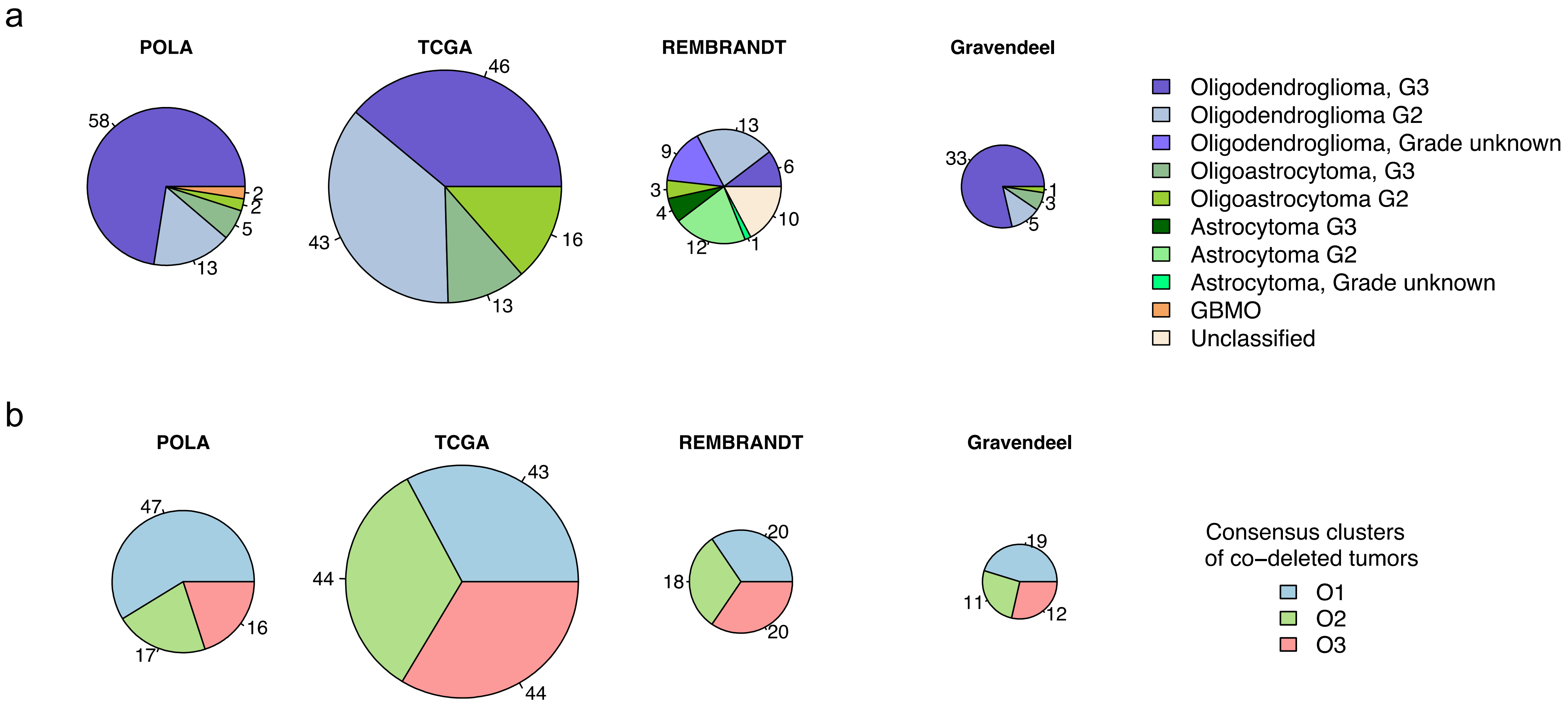

Supplementary Figure 2: Identification of O1, O2 and O3 subtypes within 3 additional public datasets of gliomas. (a): Overview of the glioma histological subtypes repartition within each cohort after selection of 1p/19q co-deleted samples only. 131, 58, and 42 1p/19q co-deleted gliomas were analysed in TCGA, REMBRANDT, and Gravendeel datasets respectively. **(b)**: Repartition of samples labelled O1, O2 and O3 in each dataset. The 3 public cohorts were each partitioned into 3 classes after unsupervised clustering analysis. O1, O2, and O3 labels were then assigned given the correlation of each class centroid with the O1, O2, and O3 class centroids computed on POLA cohort.

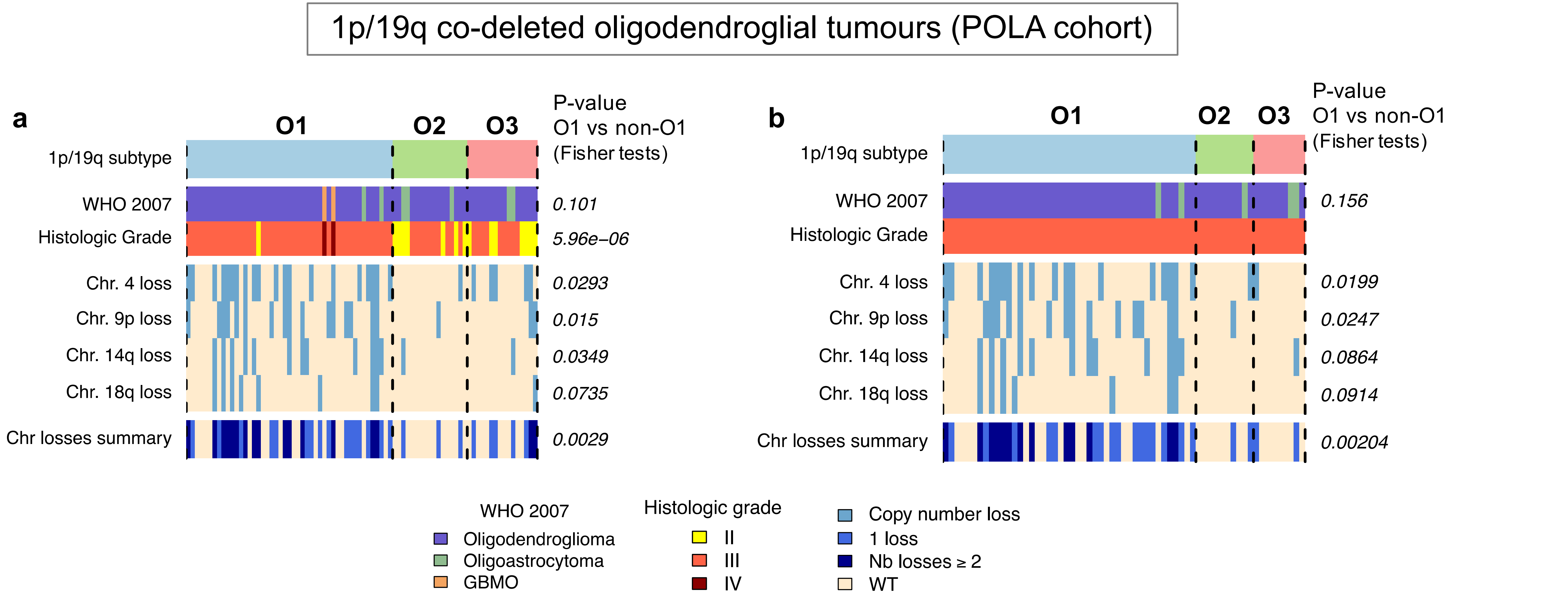

Supplementary Figure 3: Frequent genomic losses in 1p/19q co-deleted OT and their association with O1, O2, and O3 subtypes. Fisher tests were performed to measure the association of each annotation with O1 membership when considering all OT with 1p/19q co-deletion **(a)** and when restricting the cohort to only grade III tumours **(b)**. "Chr losses summary" refers to the presence of at least one chromosomal loss amongst the 4 arms/chromosomes frequently lost in O1 tumours.

1p/19q co-deleted oligodendroglial tumours (TCGA cohort)

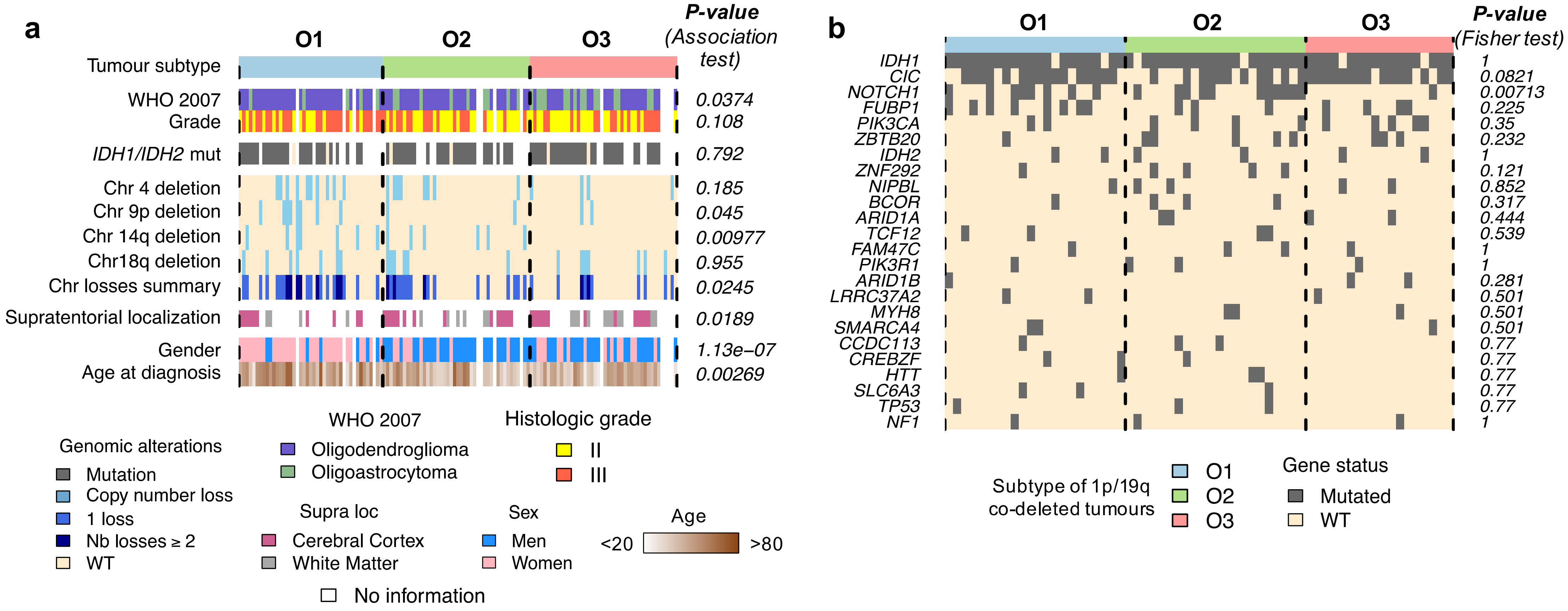

Supplementary Figure 4: Analysis of molecular and clinical data associated to TCGA O1, O2 and O3 subtypes. (a): Clinical annotations and frequent genomic alterations associated to each of the 3 tumour subtypes in either POLA discovery cohort or the TCGA cohort of 1p/19q co-deleted samples. We performed statistical tests for each variable to assess the strength of association with the 3-class partition (Fisher tests for categorical variables and Kruskal-Wallis tests for continuous variables) and displayed the corresponding p-values on the right. **(b)** Visualisation of TCGA gene mutation data based on O1/O2/O3 tumour partition. Exome data was available for 62 of the 1p/19q co-deleted TCGA samples. The panel is restricted to the genes most significantly mutated in TCGA cohort (MutSig p-value < 0.05) and which are mutated in at least 2 patients with 1p19q co-deleted tumours. Fisher tests were performed to assess the strength of association of each gene mutation profile with one of the O1, O2, O3 subtypes.

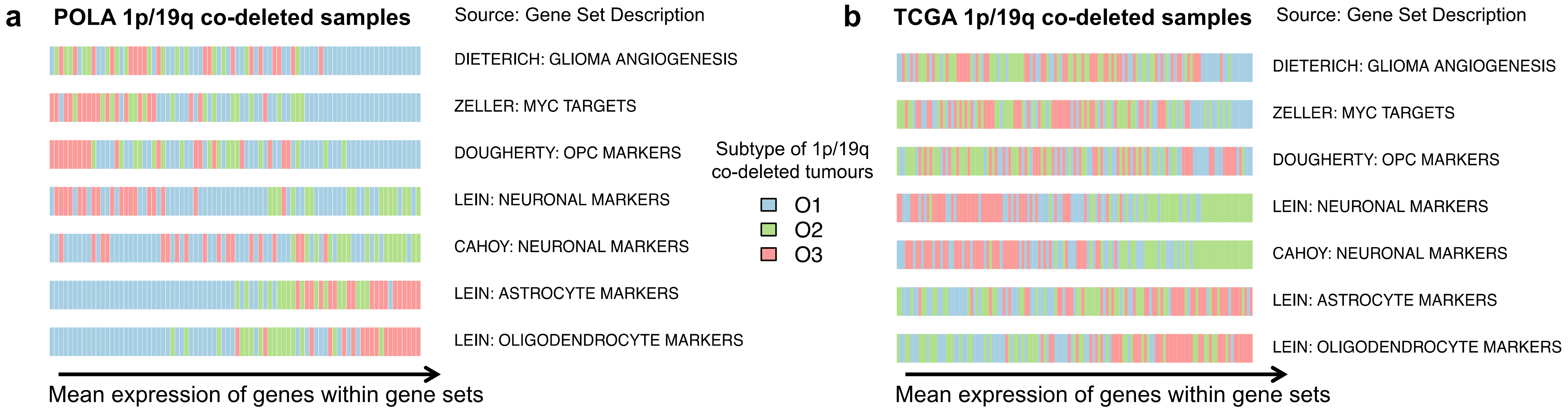

Supplementary Figure 5: Significantly deregulated gene sets in POLA 1p/19q co-deleted samples and visualisation of their activity in both POLA and TCGA co-deleted samples. (a) Visualisation of POLA 1p/19q co-deleted samples (n=80) ranked according to their mean expression value of genes within gene sets found to be significantly deregulated between subtypes (gene set enrichment analysis (GSA); Supplementary Table 2). Each row corresponds to the gene set listed on the right, and each rectangle corresponds to a tumour with a colour indicating its subtype membership (O1, O2, O3). For a given gene set, samples with the highest global expression of all the genes in the gene set are on the right hand side. Reciprocally, samples with the lowest global expression of all the genes in the gene set are on the left hand side. **(b)** TCGA 1p/19q co-deleted samples (n=131) ordered by gene sets activity as shown for POLA samples.

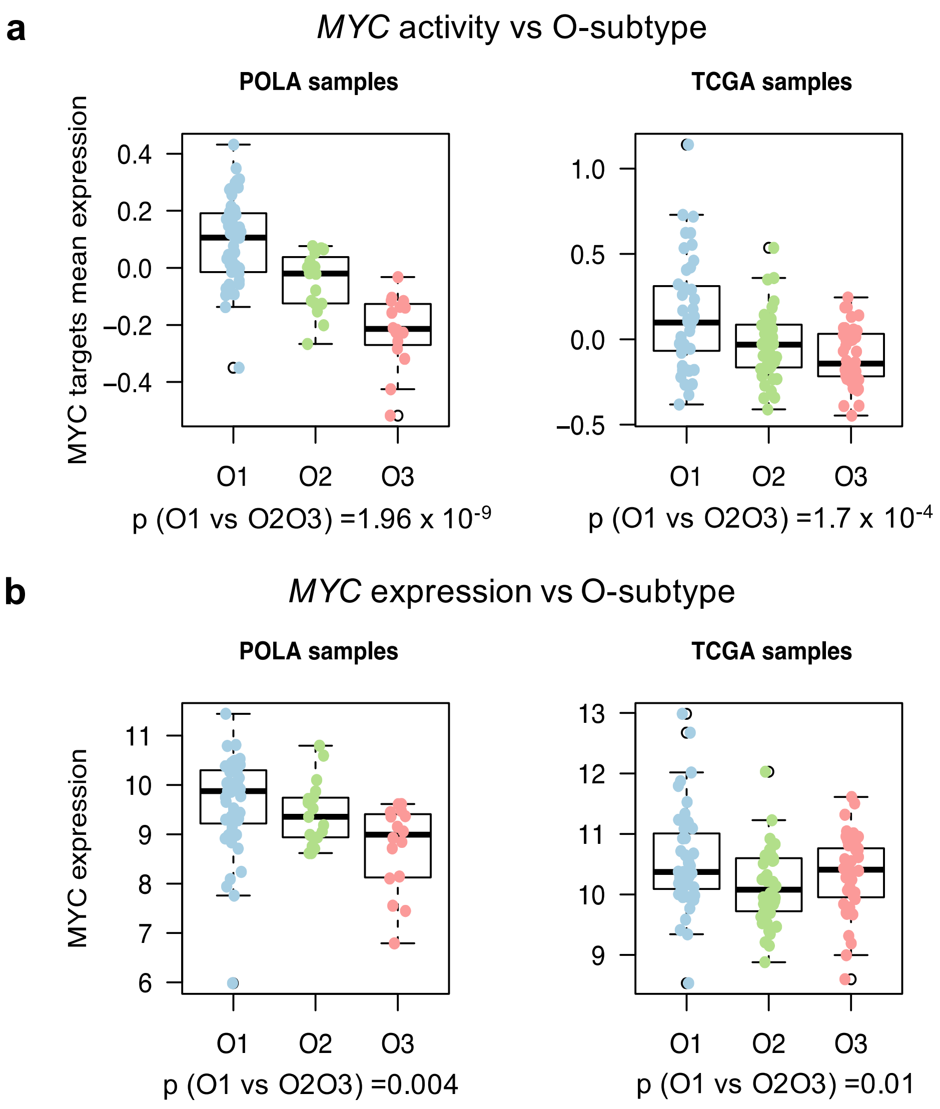

Supplementary Figure 6: *MYC* activity and *MYC* expression in 1p19q co-deleted tumour subgroups. (a) *MYC* activity is measured for each sample as the mean mRNA expression of *MYC* targets, in both POLA (80 samples) and TCGA (131 samples) data sets. We performed a t-test between O1 tumours and non-O1 tumours to assess *MYC* differential activity level in each dataset. **(b)** *MYC* expression as measured by mRNA expression arrays in POLA dataset, and RNA-seq data in TCGA data set. We performed a t-test between O1 tumours and non-O1 tumours to assess *MYC* differential expression level in each dataset. For each condition bottom and top of the boxes are the first and third quartile of the data and whiskers represent the lowest (respectively highest) data point still within 1.5 interquartile range of the lower (respectively upper) quartile. Bold lines represent median values.

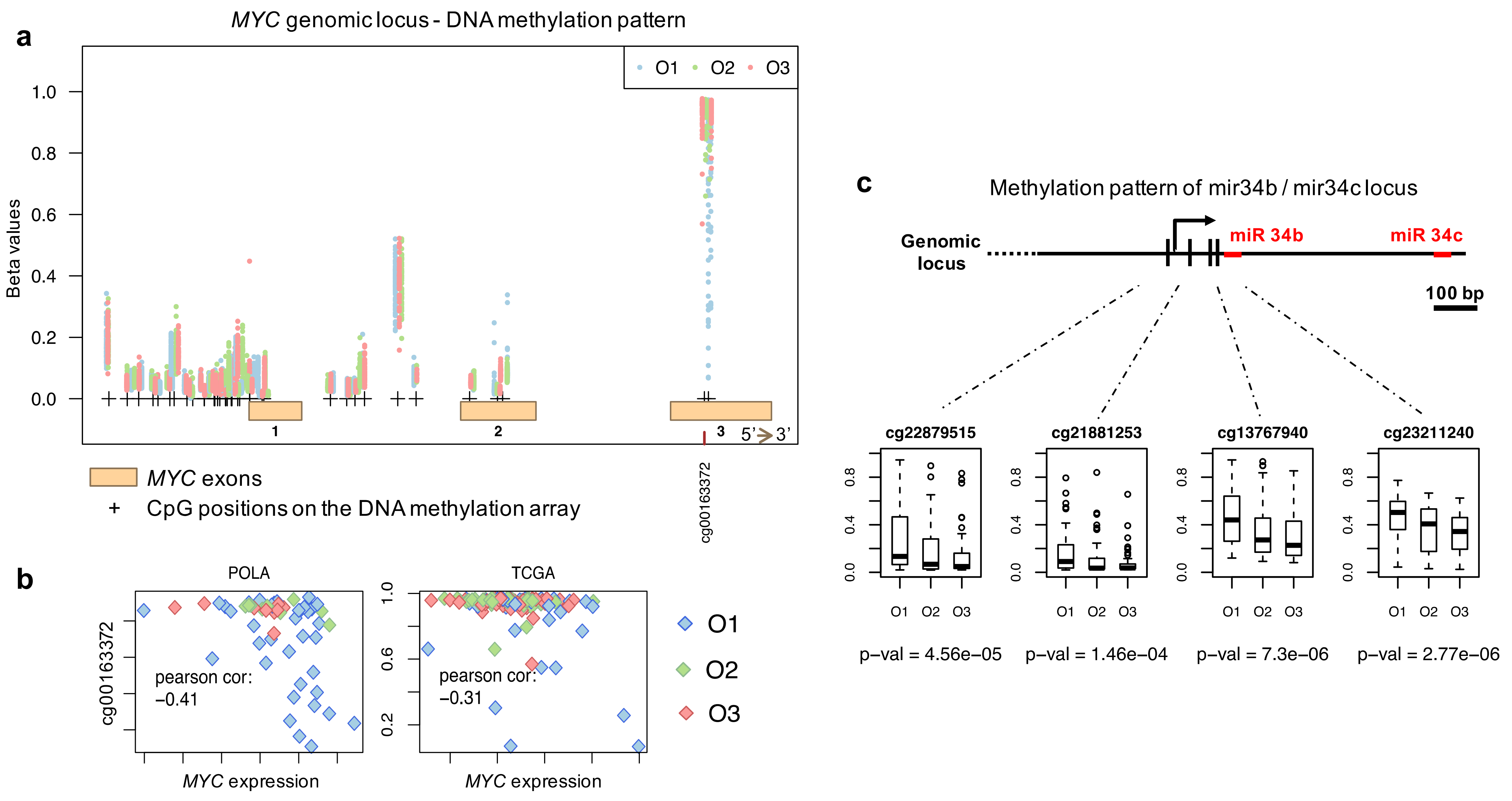

Supplementary Figure 7: Alteration of *MYC* signaling at the epigenetic level. (a) *MYC* DNA methylation profile from both POLA and TCGA methylation arrays. Black crosses indicate the positions of CpG positions targeted by the array relatively to the 3 exons of *MYC* canonical transcript (NM_002467). Distributions of samples beta values are plotted at each CpG position. Each sample data point is coloured according to O1, O2, O3 subtypes. **(b)** Relation between *MYC* mRNA expression and *MYC* exon 3 (cg00163372) hypomethylation level in each dataset. **(c)** Levels of DNA methylation at 4 CpG positions around the mir34b/34c locus transcription start site (marked with a black arrow). Red lines indicate the position of the

mature microRNAs. Wilcoxon rank sum tests were performed for each CpG position to assess differential methylation levels between O1 tumours and non-O1 tumours. For each box and whiskers plot, bottom and top of the boxes are the first and third quartile of the data and whiskers represent the lowest (respectively highest) data point still within 1.5 interquartile range of the lower (respectively upper) quartile. Bold lines represent median values. Any data not included between the whiskers are plotted as small circles.

Overall survival of 1p/19q co-deleted tumours in TCGA cohort

a

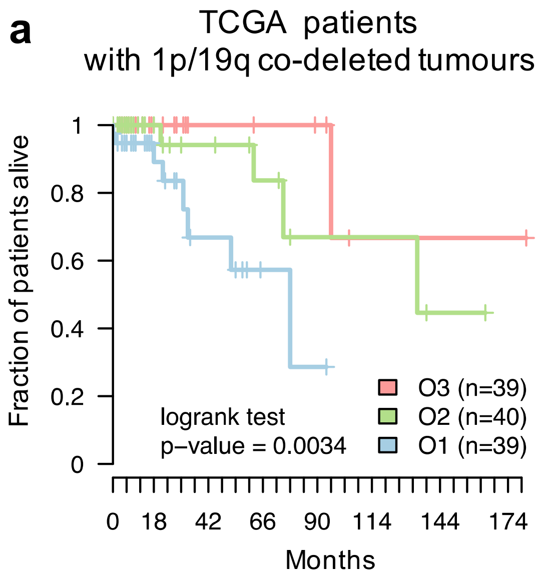

b

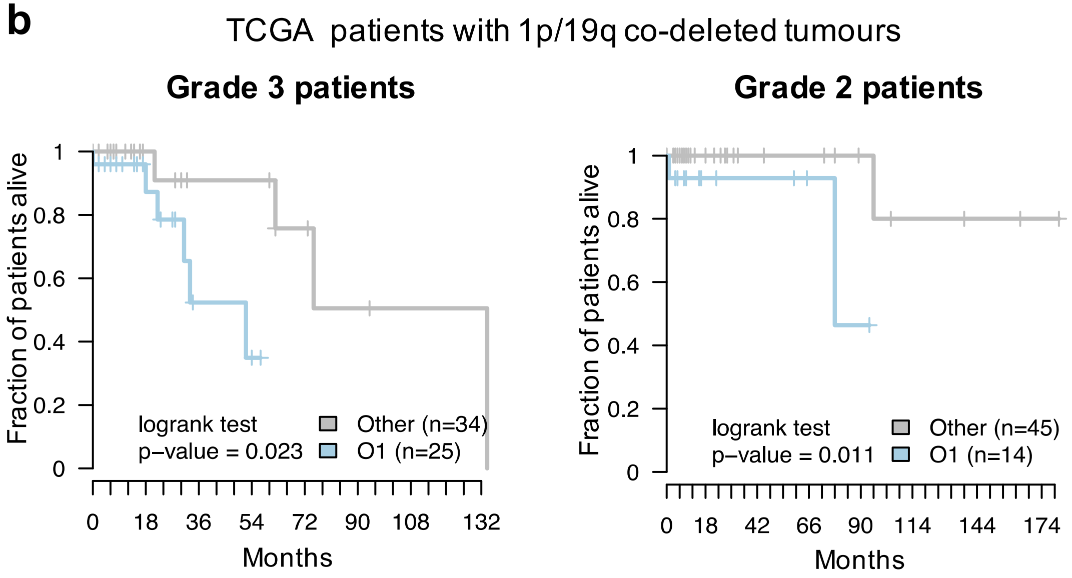

c Univariate Cox regression (TCGA)

Factor	Level	N	H.R.	95%C.I.	p-val (Wald)	p-val (log rank)
Oclass	O2	118	3,02	0,33-28	0,33	3,40E-03
**Oclass**	**O1**	**118**	**15**	**1,7-140**	**0,016**	**3,40E-03** *
**Grade**	**G3**	**118**	**6,45**	**1,7-24**	**0,0059**	**2,00E-03** *
**Age**	**Age**	**118**	**1,15**	**1,1-1,2**	**7,40E-05**	**6,70E-06** *
Gender	Female	118	0,858	0,29-2,6	0,78	7,80E-01
Histology	Oligoastrocytoma	118	1,10E-08	0-Inf	1	1,60E-01

d Multivariate Cox regression model (TCGA) : *Oclass+grade+age*

Factor	Level	N	H.R.	95%C.I.	p-val (Wald)	p-val (log rank)
Oclass	O2	118	8,24	0,69-98	0,095	3,90E-06 *
Oclass	O1	118	13,9	1,2-160	0,033	3,90E-06 *
Grade	G3	118	5,93	1,2-29	0,028	3,90E-06 *
Age	Age	118	1,15	1,1-1,3	1,40E-03	3,90E-06 *

Supplementary Figure 8: Cox univariate and multivariate regression analysis of TCGA overall survival data for 1p/19q co-deleted tumours. (a) Overall survival of TCGA patients with 1p/19q co-deleted tumours stratified by subtype. **(b)** Overall survival of TCGA patients with 1p/19q co-deleted tumours according to O1 subtype membership and histologic tumour grading. We used the available clinical data from 118 patients with co-deleted tumours (59 with grade III tumours and 59 with grade II tumours). **(c)** Results from univariate Cox regression analysis of survival data performed on each factor independently. We evaluate the statistical significance of potential predictive factor for survival: the 3-classes partition of 1p/19q co-deleted tumours (Oclass), tumour histological grade (Grade), patient age (Age), patient gender (Gender), tumour histological type (Histology). **(d)** Results from Cox regression multivariate analysis based on the model including only factors that were statistically significant in the univariate analysis presented in (a), i.e. molecular subtyping (Oclass), tumour grade (Grade) and patient age (Age). The "Level" column refers to the factor level which is been tested as prognosis factor relatively to the reference level of this factor. References levels are: O3 molecular subtype for the "Oclass" factor, G2 histological grade for the "Grade" factor, male patients for the "Gender" factor, and oligodendroglioma histological subtype for the "Histology" Factor. The "N" column gives the number of patients with complete available information for the factors tested. Hazard ratios and corresponding confidence intervals and Wald test p-values are given for each level of each factor. Log rank test p-values refer to the model considered (univariate or multivariate).

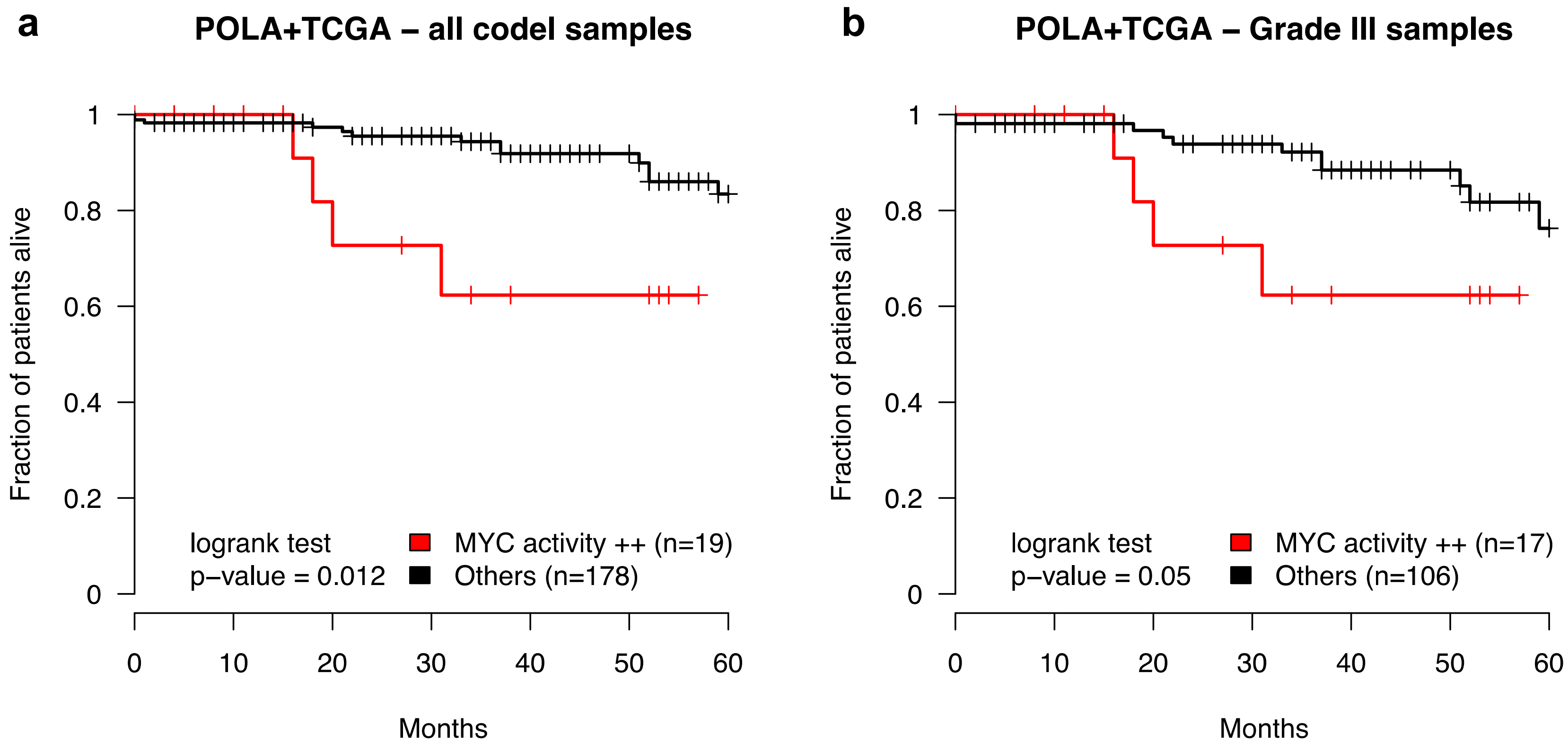

Supplementary Figure 9: Overall survival of 1p19q co-deleted patients with the highest *MYC* activity. Patients with highest *MYC* activity were defined as follow for each dataset independently (POLA and TCGA): mean expression of *MYC* targets > [mean value + 1.5*standard deviation]. Their survival was then compared to the other 1p/19q co-deleted patients, in the pooled cohort POLA+TCGA. **(a)** Overall survival among all 197 patients with 1p/19q co-deleted tumours **(b)** Overall survival restricted to grade III patients with 1p/19q co-deleted tumours (123 patients).

Sample Name	Gender	Age at surgery	Histologic Grade	WHO2007	Codel 1p19q	IDH1 mut	IDH2 mut	CIC mut	hTERT mut	TP53 IHC	ATRX IHC	Tumour Cellularity	Necrosis detected	Microvascular Proliferation	Seizure at diagnosis	Initial Radiotherapy	Death	Follow-up (months)	Transcriptome Profiling	Methylome Profiling	Mirnome Profiling	SNP Profiling	Verhaak subtype	Gravendeel subtype	Cluster of clusters	Codel subtype
HS_1077	F	40	2	Oligodendroglioma	1	M	WT	WT	M	0			N	N	Y	N	0	50	Y	N	Y	N	Pro	9	C3	O2
HS_1237	M	33	2	Oligodendroglioma	1	WT	WT			0			N	N	Y	N	0	43	Y	N	Y	Y	Neu	0		
HS_1585	M	42	3	Diffuse astrocytoma	0	M	WT			1			N	N	Y	N	1	76	Y	N	Y	N	Neu	17	C5	
HS_1598	M	47	2	Oligodendroglioma	0	M	WT		WT	1			N	N	Y		0	90	Y	N	Y	N	Mes	17	C4	
HS_1630	M	36	2	Oligoastrocytoma	0	M	WT		WT	1			N	N	Y	N	0	30	Y	N	Y	Y	Mes	17	C5	
HS_1690	F	44	2	Oligoastrocytoma	0	M	WT			1			N	N	N	N	0	21	Y	N	Y	N	Pro	17	C4	
HS_1773	M	25	2	Oligodendroglioma	1	WT	M	WT		0		0,81	N	N	Y		0	56	Y	N	Y	Y	Neu	9	C3	O2
HS_1788	F	35	2	Oligoastrocytoma	1	M	WT	WT	M	0			N	N	Y	N	0	28	Y	N	Y	Y	Neu	0		
HS_1955E				Normal											Y				Y	N	Y	N	Neu	0		
HS_1970	M	47	2	Oligoastrocytoma	0	M	WT		WT	1		0,75	N	N			0	17	Y	N	Y	N	Neu	17	C4	
HS_2090E				Normal											Y				Y	N	Y	N	Neu	0		
HS_2095E				Normal											Y				Y	N	Y	N	Neu	0		
HS_2145	F	41	3	Diffuse astrocytoma	0	M	WT			1		0,6	N	N	N	N	0	85	Y	N	Y	Y	Mes	17	C4	
HS_2169	M	56	2	Oligoastrocytoma	1	M	WT	WT		0			N	N		N			Y	N	Y	Y	Pro	9	C3	O2
HS_2244	F	34	2	Oligodendroglioma	1	M	WT	WT		0			N	N	Y	N	0	80	Y	N	Y	Y	Pro	17	C5	O3
HS_2256	F	66	2	Oligoastrocytoma	0	WT	WT		M	1			N	N		N	0	8	Y	N	Y	N	Pro	17	C4	
HS_2292	F	47	2	Oligoastrocytoma	0	M	WT		WT	1		0,88	N	N		N	0	5	Y	N	Y	Y	Neu	17	C4	
HS_2306	F	28	2	Oligoastrocytoma	0	M	WT		WT	1			N	N		N	0	62	Y	N	Y	Y	Neu	0		
HS_2382	F	45	2	Oligodendroglioma	0	M	WT	WT	WT	0			N	N		N	1	58	Y	N	Y	N	Pro	17	C4	
HS_2390	F	26	2	Oligoastrocytoma	0	WT	WT		WT	0			N	N	Y	N	0	74	Y	N	Y	N	Neu	17	C5	
HS_2391	M	32	2	Oligoastrocytoma	1	M	WT	M	M	0			N	N	Y	N	0	55	Y	N	Y	N	Pro	9	C3	O2
HS_2402	M	58	3	Oligodendroglioma	1	M	WT	WT	M	0	1	0,26	N	Y	Y	Y	1	35	Y	Y	Y	Y	Neu	9		
HS_2425	M	46	3	Oligodendroglioma	1	M	WT	WT	M	0	1	0,75	Y	Y	Y	Y	0	63	Y	N	Y	Y	Mes	17	C5	O3
HS_2441	M	49	3	Oligodendroglioma	0	WT	WT	WT	WT	1		0,4	Y	Y	N	Y	1	54	Y	Y	Y	Y	Mes	18	C2	
HS_2463	F	44	3	Oligodendroglioma	0	WT	WT			0	0	0,25	Y	Y	Y	Y	1	50	Y	N	Y	Y	Mes	18	C2	
HS_2464	M	62	3	Oligodendroglioma	1	M	WT		M	1	1	0,43	N	Y	N	Y	0	9	Y	Y	Y	Y	Neu	17	C5	O3
HS_2468	M	31	2	Oligoastrocytoma	0	M	WT			1		0,95	N	N	Y	N	1	44	Y	N	Y	Y	Pro	17	C4	
HS_2472	F	60	3	Oligodendroglioma	1	M	WT	M	M	1	1	0,92	N	Y	N	Y	1	59	Y	Y	Y	Y	Pro	9	C1	O1
HS_2483	F	51	3	Oligoastrocytoma	0	M	WT		WT	1	0		N	Y	N	N	1	1	Y	Y	Y	Y	Pro	17	C4	
HS_2485	M	61	3	Oligodendroglioma	1	M	WT		M	0	1	0,95	N	N	Y	Y	0	62	Y	N	Y	Y	Pro	9	C1	O1
HS_2486	M	32	3	Oligodendroglioma	1	M	WT	WT	M	0	1	0,9	Y	Y	Y	Y	0	61	Y	N	Y	Y	Pro	9	C3	O2
HS_2496	M	42	3	Oligodendroglioma	1	M	WT	M	M	0	1	0,8	N	Y	N	N	0	66	Y	Y	Y	Y	Pro	9	C1	O1
HS_2532	F	42	3	Oligodendroglioma	1	M	WT	WT	M	0	1	0,5	Y	Y	N	Y	0	67	Y	Y	Y	Y	Mes	9	C1	O1
HS_2537	F	67	3	Oligodendroglioma	0	WT	WT		WT	0	1	0,95	N	Y	Y	Y	1	20	Y	Y	Y	Y	Neu	0		
HS_2543	M	75	3	Oligodendroglioma	1	M	WT	M	M	0	1	0,9	N	Y	N	N	1	37	Y	Y	Y	Y	Pro	9	C1	O1
HS_2578	M	42	2	Oligodendroglioma	1	M	WT	WT		0		0,62				N	0	30	Y	N	Y	Y	Neu	0		
HS_2593	M	39	2	Oligoastrocytoma	0	M	WT		WT	1		0,8	N	N	Y	N	0	67	Y	N	Y	Y	Neu	17	C4	
HS_2612	M	56	4	GBMO	0	WT	WT			1		0,88	Y	Y	Y	Y	1	19	Y	Y	Y	Y	Cla	18	C2	
HS_2618	F	28	3	Oligodendroglioma	1	M	WT	M	M	0	0	0,6	Y	Y	N	Y	0	57	Y	Y	Y	Y	Mes	9	C1	O1
HS_2619	M	34	3	Oligodendroglioma	0	WT	WT		WT	1		0,8	N	Y	Y	Y	1	34	Y	Y	Y	Y	Pro	9	C2	
HS_2622	F	28	2	Oligodendroglioma	0	M	WT		WT								0	0	Y	N	Y	Y	Mes	17	C4	
HS_2624	F	38	2	Oligodendroglioma	1	M	WT	WT	M	0		0,72					0	19	Y	N	Y	Y	Neu	17		
HS_2626	M	58	3	Oligodendroglioma	1	M	WT	WT	M	0	0	0,91	N	Y	N	Y	0	61	Y	Y	Y	Y	Pro	9	C1	O1
HS_2646	M	63	4	Glioblastoma	0							0,1				Y	0	28	Y	N	Y	Y	Mes	23	C5	
HS_2661	F	54	3	Oligodendroglioma	1	M	WT	M		0	1	0,87	N	Y	Y	Y	0	63	Y	Y	Y	Y	Pro	9	C3	O2
HS_2668	M	37	3	Oligodendroglioma	1	WT	M	M	M	0	1	0,95	N	Y	Y	Y	0	54	Y	Y	Y	Y	Pro	9	C1	O1
HS_2669	M	34	3	Oligodendroglioma	1	M	WT	WT	M	0		0,26			N	N	0	57	Y	Y	Y	Y	Neu	0		
HS_2671	M	45	3	Oligodendroglioma	1	M	WT	M	M	0	1	0,9	N	Y	N	Y	0	36	Y	Y	Y	Y	Pro	9	C1	O1
HS_2673	M	30	2	Oligoastrocytoma	0	M	WT		M	1		0,91					0	24	Y	N	Y	Y	Neu	17	C4	
HS_2691	M	46	3	Oligodendroglioma	1	M	WT		M	0	1	0,66	N	Y	Y	Y	0	57	Y	N	Y	Y	Mes	9	C1	O1
HS_2694	F	51	3	Oligodendroglioma	1	WT			M	0	1	0,95	Y	Y	N	Y	1	51	Y	Y	Y	Y	Pro	9	C1	O1
HS_2695	M	30	3	Oligoastrocytoma	0	M	WT			1	0	0,63	N	Y	N	Y	0	50	Y	Y	Y	Y	Mes	17	C2	
HS_2702	M	61	3	Oligodendroglioma	1	M	WT	M	M	0	1	0,91	Y	Y	N	Y	1	20	Y	Y	Y	Y	Pro	9	C1	O1
HS_2708	F	53	3	Oligodendroglioma	1	M	WT		M	0	1	0,3	N	Y	Y	Y	0	55	Y	Y	Y	Y	Neu	0		
HS_2709	M	61	3	Oligodendroglioma	0	WT	WT			1		0,1	Y	Y	N	Y	1	13	Y	Y	Y	Y	Neu	0		
HS_2715	M	55	4	GBMO	0	WT	WT			1	1	0,76	Y	Y	N	Y	1	12	Y	Y	Y	Y	Mes	18	C2	
HS_2716	F	40	3	Oligodendroglioma	1	M	WT	M	M	1	1	0,91	N	N	N	Y	0	58	Y	Y	Y	Y	Pro	9	C1	O1
HS_2728	F	35	3	Oligodendroglioma	1	M	WT	WT	WT	0	1	0,22	N	N	Y	Y	0	57	Y	Y	Y	Y	Pro	9	C5	O3
HS_2753	F	37	4	Glioblastoma	0	WT	WT			0	1	0,85	N	Y	Y	Y	1	20	Y	Y	Y	Y	Cla	18	C2	
HS_2754	M	42	3	Oligodendroglioma	1	M	WT	M	M	0	1	0,93	N	Y	Y	Y	0	53	Y	Y	Y	Y	Pro	9	C1	O1
HS_2755	M	60	3	Oligodendroglioma	1	M	WT	M	M	0	1	0,92	N	Y	Y	Y	0	54	Y	Y	Y	Y	Pro	9	C3	O2

HS_2775	M	46	3	Oligodendroglioma	1	M	WT		M	0	1	0,4	N	Y	N	Y	0	57	Y	N	Y	Y	Pro	9	C1	O1
HS_2785	M	39	3	Oligodendroglioma	1	M	WT	M	M	0	1		Y	Y	Y	Y	0	40	Y	Y	Y	N	Pro	9	C1	O1
HS_2786	F	48	3	Oligodendroglioma	0	M	WT		WT	1		0,9	N	Y	N	Y	0	32	Y	Y	Y	Y	Mes	17	C4	
HS_2794	M	72	2	Oligodendroglioma	1	M	WT	M		0		0,89	N	N	N	Y	0	4	Y	Y	Y	Y	Pro	9	C3	O1
HS_2795	M	36	3	Oligodendroglioma	1	M	WT	M	M	0	1	0,84	Y	Y	N	Y	1	37	Y	Y	Y	Y	Pro	9	C1	O1
HS_2807	F	35	3	Oligodendroglioma	1	WT	M			0	1	0,7	N	Y	Y	Y	0	51	Y	Y	Y	Y	Pro	9	C1	O1
HS_2821	F	56	3	Oligodendroglioma	1	M	WT	WT	M	0	1	0,9	N	Y	Y	N	0	59	Y	Y	Y	Y	Neu	9		
HS_2826	F	47	3	Oligodendroglioma	1	M	WT	WT			1	0,8	N	Y	N	Y	0	10	Y	N	Y	Y	Mes	17	C1	O1
HS_2828	M	23	3	Oligodendroglioma	0	M	WT			1	1	0,4	N	Y	Y	Y	0	34	Y	N		Y	Cla	18	C2	
HS_2830	M	64	3	Oligodendroglioma	1	M	WT	M	M	0	1	0,78	N	Y	N	Y	0	50	Y	Y	Y	Y	Mes	9	C1	O1
HS_2832	M	63	3	Oligodendroglioma	1	M	WT	M	M			0,89	N	Y	Y	N			Y	Y	Y	Y	Pro	9	C1	O1
HS_2840	M	24	3	Oligodendroglioma	1	M		WT		0	0	0,79	N	N	Y	Y	0	41	Y	Y	Y	Y	Neu	17	C3	O2
HS_2842	F	55	3	Oligodendroglioma	1	M	WT	WT	M	0	1	0,94	N	Y	N	Y	0	44	Y	Y	Y	Y	Pro	9	C1	O1
HS_2847	F	49	3	Oligodendroglioma	0	WT	WT			0	1	0	N	Y	Y	N	0	55	Y	N	Y	Y	Neu	0		
HS_2848	F	62	3	Oligodendroglioma	1	M	WT		WT	1	1	0,5	N	Y	Y	Y	0	55	Y	N	Y	Y	Mes	17		
HS_2855	M	62	4	GBMO	0	WT	WT			0	1	0,72	Y	Y	Y	Y	1	10	Y	Y	Y	Y	Mes	18	C2	
HS_2865	F	64	3	Oligoastrocytoma	0	M	WT			1			N	Y	Y	Y	1	37	Y	N	Y	Y	Mes	22	C2	
HS_2866	F	45	3	Oligodendroglioma	1	M	WT	WT	M	0	1	0,5	N	Y	N	Y	0	46	Y	Y	Y	Y	Pro	17	C5	O3
HS_2877	M	78	3	Oligodendroglioma	1	M	WT		M	1	1	0,7	Y	Y	N	Y	1	22	Y	Y	Y	Y	Neu	17	C3	O2
HS_2878	M	53	3	Oligodendroglioma	1	M	WT	M	M	0	1	0,95	N	Y	N	Y	0	38	Y	Y	Y	Y	Pro	9	C1	O1
HS_2890	M	27	3	Oligodendroglioma	0	WT	WT			0	1	0,9	Y	Y	N	Y	1	15	Y	Y	Y	Y	Pro	22	C2	
HS_2895	M	42	3	Oligoastrocytoma	0	M	WT			0	0	0,85	N	Y	N	Y	0	34	Y	N	Y	Y	Mes	17	C4	
HS_2896	M	78	3	Oligodendroglioma	1	M	WT		WT	0	1	0,95	Y	Y		N	1	0	Y	Y	Y	Y	Pro	9	C1	O1
HS_2897	F	47	3	Oligodendroglioma	1	M	WT	WT		0	1	0,9	N	Y	Y	Y	0	34	Y	Y	Y	Y	Pro	9	C1	O1
HS_2898	M	33	3	Oligodendroglioma	1	WT	M	WT	M	0	1	0,6	N	N	Y	Y	0	54	Y	Y	Y	Y	Neu	17		
HS_2911	M	47	3	Oligodendroglioma	1	M	WT	M	M	0	1	0,94	N	Y	N	Y	0	38	Y	Y	Y	Y	Pro	9	C1	O1
HS_2922	M	44	2	Oligoastrocytoma	0	M	WT		WT	1							0	0	Y	N	Y	Y	Neu	17	C4	
HS_2935	M	49	4	GBMO	0	WT	WT			1		0,83	Y	Y	N	Y	0	2	Y	Y	Y	Y	Cla	18	C2	
HS_2936	F	52	4	Glioblastoma	0	WT	WT			0		0,89	Y	Y	Y	Y	1	40	Y	Y	Y	Y	Cla	18	C2	
HS_2937	M	76	4	Glioblastoma	0	WT	WT			1	0	0,9	Y	Y	Y	Y	1	11	Y	N	Y	Y	Pro	22	C2	
HS_2941	F	35	3	Oligoastrocytoma	0	M	WT			1	0	0,63	N	Y	Y	Y	0	49	Y	Y	Y	Y	Pro	17	C4	
HS_2943	M	41	4	Glioblastoma	0	WT	WT			0		0,6	Y	Y	Y	Y	0	41	Y	Y	Y	Y	Mes	18	C2	
HS_2944	F	58	3	Oligoastrocytoma	0	WT	WT			1	0	0,6	N	Y	Y	Y	1	17	Y	Y	Y	Y	Mes	17	C4	
HS_2951	F	61	2	Oligodendroglioma	1	M	WT	M		1		0,76	N	N		N	0	30	Y	Y	Y	Y	Mes	17	C5	O3
HS_2952	M	40	3	Oligodendroglioma	1	M	WT	WT	M	0	1	0,7	N	N	Y	N	0	29	Y	Y	Y	Y	Neu	17		
HS_2953	F	61	3	Oligodendroglioma	1	M	WT	WT		0	1	0,95	N	Y		N	0	36	Y	Y	Y	Y	Pro	9	C1	O1
HS_2965	F	29	3	Oligodendroglioma	1	M	WT	WT	M	0	1	0,95	Y	Y		N	0	53	Y	Y	Y	Y	Pro	9	C1	O1
HS_2971	M	31	3	Oligodendroglioma	1	M	WT	M	M	0	1	0,5	N	Y	Y	Y	0	49	Y	Y	Y	Y	Neu	0		
HS_3000	M	34	3	Oligodendroglioma	1	WT	M	M	M	0	1	0,95	Y	Y	Y	Y	0	52	Y	Y	Y	Y	Pro	9	C1	O1
HS_3007	F	31	4	GBMO	0	M	WT			1	0		Y	Y	N	Y	1	19	Y	Y	Y	Y	Cla	17	C4	
HS_3018	M	56	3	Oligodendroglioma	1	WT	M			1	1	0,95	Y	Y	Y	Y	1	16	Y	N	Y	Y	Pro	9	C1	O1
HS_3020	M	40	2	Oligodendroglioma	1	M	WT	M		0		0,4	N	N	Y	Y	0	50	Y	Y	Y	Y	Neu	0		
HS_3032	F	51	3	Oligodendroglioma	1	M	WT	M	M	0	1	0,85	N	Y	Y	Y	0	32	Y	Y	Y	Y	Pro	9	C3	O2
HS_3059	M	59	4	GBMO	0	WT	WT			1	0	0,5	Y	Y	N	Y	1	11	Y	Y	Y	Y	Neu	0		
HS_3060	F	35	4	Glioblastoma	0	M	WT			1	0	0,95	N	Y	N	Y	0	25	Y	Y	Y	Y	Pro	22	C2	
HS_3061	F	19	4	Glioblastoma	0	WT	WT			1	0	0,95	Y	Y	N	N	1	2	Y	Y	Y	Y	Pro	22	C2	
HS_3062	F	57	4	GBMO	1	M	WT	WT		0	1	0,85	Y	Y	N	Y	0	41	Y	Y	Y	Y	Pro	9	C1	O1
HS_3063	F	45	3	Oligodendroglioma	1	M	WT	M	WT	0	1	0,7	N	Y	Y	Y	0	46	Y	Y	Y	Y	Pro	17	C1	O1
HS_3064	F	51	3	Oligodendroglioma	1	M	WT			0	1	0,7	N	Y	N	Y	1	18	Y	Y	Y	Y	Neu	9	C3	O2
HS_3067	F	58		NOS	0	WT	WT			1			N	Y	N	Y	1	23	Y	N	Y	Y	Cla	18	C2	
HS_3068	F	27	4	GBMO	1	M	WT	WT		0	1	0,95	Y	Y		Y	0	24	Y	N	Y	Y	Pro	9	C1	O1
HS_3074	M	38	2	Oligodendroglioma	1	M	WT	WT	M	0		0,7			Y	N	0	11	Y	N	Y	Y	Neu	17	C3	O2
HS_3093	F	40	3	Oligodendroglioma	0	WT	WT			0	1	0,7	Y	Y	Y	Y	0	38	Y	Y	Y	Y	Cla	18	C2	
HS_3096	F	55	2	Oligodendroglioma	1	M	WT	WT	M	0		0,9					0	9	Y	N	Y	Y	Neu	17	C4	O3
HS_3122	F	34	3	Oligodendroglioma	1	M	WT		M	0	1	0,9	N	N	Y	Y	0	52	Y	Y	Y	Y	Pro	9	C1	O1
HS_3125	F	38	3	Oligodendroglioma	1	M	WT	M		0	1	0,94	Y	Y	N	Y	0	38	Y	Y	Y	Y	Pro	9	C1	O1
HS_3126	F	45	3	Oligodendroglioma	1	M	WT	WT	WT	0	1	0,3	N	Y		Y	1	12	Y	Y	Y	Y	Neu	0		
HS_3130	M	54	3	Oligodendroglioma	0	WT	WT		WT	0		0,6	N	Y	Y	Y	1	41	Y	Y	Y	Y	Pro	22	C2	
HS_3132	M	18	2	Oligodendroglioma	0	M	WT			0		0,2					0	8	Y	N	Y	Y	Mes	17	C4	
HS_3142	M	56	3	Oligodendroglioma	1	M	WT			0	1	0,9	N	Y	Y	Y	0	47	Y	N	Y	Y	Pro	9	C1	O1
HS_3144	M	62	4	Glioblastoma	0	WT	WT			0		0	Y	Y		N	0	35	Y	Y	Y	Y	Neu	0		
HS_3149	M	61	3	Oligodendroglioma	1	M	WT	WT	M	0	1	0,57	N	N	Y	Y	0	8	Y	Y	Y	Y	Pro	9	C1	O1
HS_3237	M	46	3	Oligodendroglioma	1	M	WT		WT	1	0	0,7	N	Y	Y	Y	0	43	Y	N	Y	Y	Mes	9	C1	O1

HS_3240	M	29	3	Oligodendroglioma	0	M	WT			1	0	0,6	N	N		N	1	3	Y	Y	Y	Y	Pro	22	C2	
HS_3245	M	44	3	Oligodendroglioma	1	M	WT	M	M	1	1	0,51	Y	Y	N	Y	0	46	Y	Y	Y	Y	Pro	9	C1	O1
HS_3254	M	63	4	Glioblastoma	0	WT	WT			0	1	0,88	Y	Y	Y	Y	0	44	Y	Y	Y	Y	Cla	18	C2	
HS_3258	F	52	3	Oligodendroglioma	1	M	WT	WT	M	0	1	0,81	N	N		N	0	43	Y	Y	Y	Y	Neu	0		
HS_3270	M	28	3	Oligoastrocytoma	1	M	WT			0	1	0,95	N	Y	Y	Y	0	42	Y	Y	Y	Y	Pro	9	C1	O1
HS_3318	M	61	4	Glioblastoma	0	WT	WT			0	1	0,86	N	Y		Y	0	40	Y	Y	Y	Y	Cla	18	C2	
HS_3319	M	59	3	Oligoastrocytoma	0	M	WT			1	1	0,67	N	Y		Y	0	39	Y	Y	Y	Y	Mes	17	C2	
HS_3320	M	36	3	Oligoastrocytoma	1	M	WT		M	0	1	0,5	N	Y		Y	0	36	Y	Y	Y	Y	Neu	17		
HS_3327	F	45	3	Oligodendroglioma	1	M	WT	M		0	1	0,9	Y	Y		Y	0	27	Y	Y	Y	Y	Pro	9	C1	O1
HS_3334	M	49	3	Oligodendroglioma	1	M	WT	M		0	1	0,73	Y	Y		Y	0	38	Y	N	Y	Y	Pro	9	C1	O1
HS_3358	F	75	4	Glioblastoma	0	M	WT			0	1	0,84	N	Y		Y	1	13	Y	Y	Y	Y	Cla	18	C2	
HS_3359	M	41	3	Oligoastrocytoma	0	M	WT		WT	1	0	0,6	N	Y		Y	0	34	Y	Y	Y	Y	Mes	17	C4	
HS_3401	M	44	3	Oligodendroglioma	1	M	WT		WT	0	1	0,2	Y	Y		Y	0	34	Y	N	Y	Y	Neu	0		
HS_3413	M	56	3	Oligoastrocytoma	0	M	WT		WT	1	0	0,95	N			Y	0	42	Y	Y	Y	Y	Neu	0		
HS_3434	M	35	3	Oligoastrocytoma	0	M	WT			1	0	0,5	N	Y		Y	0	37	Y	Y	Y	Y	Mes	17	C4	
HS_3443	M	42	3	Oligodendroglioma	1	WT	M	WT		0	1	0,5	N	Y		Y	0	36	Y	Y	Y	Y	Neu	17	C5	O3
HS_3459	M	54	3	Oligodendroglioma	1	M	WT	WT		0	1	0,85	N	Y	Y	Y	0	39	Y	Y	Y	Y	Mes	17	C4	O3
HS_3460	F	50	3	Oligodendroglioma	1	M	WT	WT	M	0	1	0,69	Y	Y		Y	0	36	Y	Y	Y	Y	Pro	9	C1	O1
HS_3463	M	48	3	Oligoastrocytoma	1	M	WT	WT	M	0	1	0,93	N	Y	N	Y	0	39	Y	Y	Y	Y	Pro	9	C1	O1
HS_3464	F	48	3	Oligodendroglioma	1	M	WT	M		0	1	0,95	N	N		Y	0	39	Y	Y	Y	Y	Pro	9	C3	O2
HS_3472	M	56	3	Oligoastrocytoma	1	M	WT	M		1	1	0,6	N	N		Y	0	37	Y	Y	Y	Y	Mes	17	C5	O3
HS_3474	M	42	3	Oligodendroglioma	1	WT	WT	WT		0	1	0,1	N	Y		Y	0	15	Y	Y	Y	Y	Neu	0		
HS_3476	F	70	3	Oligodendroglioma	1	M	WT	M		0	1	0,89	N	Y	Y	N	0	31	Y	N	Y	Y	Pro	9	C1	O1
HS_3488	M	39	3	Oligoastrocytoma	1	M		WT		0	1	0,8	N	Y		N	0	40	Y	Y	Y	Y	Pro	9	C3	O2
HS_3497	M	49	3	Oligodendroglioma	1	M	WT	M		0	1	0,95	N	Y		N	0	37	Y	Y	Y	Y	Pro	9	C1	O1
HS_3511	M	29	2	Oligodendroglioma	1	M	WT	M		0		0,9	N	N	Y	N	0	43	Y	N	Y	Y	Pro	9	C3	O2
HS_3528	F	51	3	Oligoastrocytoma	1	WT	M	WT		0	1	0,8	N	Y		Y	0	35	Y	Y	Y	Y	Pro	17	C5	O3
HS_3529	F	28	2	Oligodendroglioma	0	WT	WT			0		0	N	Y		N	0	29	Y	Y	Y	Y	Mes	17	C5	
HS_3543	F	38	4	GBMO	0	M	WT		M	1	0	0,94	Y	Y	Y	Y	0	33	Y	Y	Y	Y	Pro	17	C4	
HS_3546	M	44	4	GBMO	0	WT	WT			0		0,9	Y	Y		Y	0	37	Y	Y	Y	Y	Mes	18	C2	
HS_3551	M	46	3	Oligodendroglioma	1	M	WT	M		0	1	0,95	N	Y		Y	0	17	Y	Y	Y	Y	Pro	9	C3	O2
HS_3552	M	33	3	Oligoastrocytoma	0	M	WT			1	0	0,5	N	N		Y	0	18	Y	Y	Y	Y	Mes	17	C5	
HS_3557	M	39	3	Oligodendroglioma	1	M	WT	M		0	1	0,95	N	N	Y	Y	0	29	Y	Y	Y	Y	Neu	17	C5	O3
HS_3558	F	39	3	Oligoastrocytoma	0	M	WT	WT		1	0	0,9	N	N	Y	Y	0	31	Y	Y	Y	Y	Pro	17	C4	
HS_3642	M	35	2	Oligodendroglioma	0	M	WT			1			N	N	Y		0	0	Y	N	Y	Y	Neu	17	C5	
HS_3644	M	51	2	Oligodendroglioma	1	M	WT			1		0,6	N	N	Y	N	0	105	Y	N		Y	Neu	17	C5	O3
HS_3645	F	49	2	Oligodendroglioma	1	M	WT			1		0,85	N	N	Y	N	0	75	Y	N	Y	Y	Pro	9	C3	O2
HS_3646	F	60	2	Oligodendroglioma	1	M	WT	WT		0		0,5	N	N	Y		1	61	Y	N	Y	Y	Neu	0		
HS_3647	F	39	2	Oligodendroglioma	1	M	WT			0		0,7	N	N	Y		0	92	Y	N	Y	Y	Mes	17	C5	O3
HS_3648	F	24	2	Oligodendroglioma	0	WT	WT			0		0	N	N	Y	N	1	40	Y	N	Y	Y	Neu	0		
HS_3649	M	37	2	Oligodendroglioma	1	M	WT			0		0,6	N	N	Y	N	1	89	Y	N	Y	Y	Pro	9	C5	O3
HS_3650	F	32	2	Oligodendroglioma	0	M	WT			1		0,7	N	N	Y	N	1	61	Y	N	Y	Y	Mes	17	C5	
HS_3651	F	53	2	Oligodendroglioma	0	WT	WT			0		0,6	N	N	Y	N	1	9	Y	N	Y	Y	Neu	17		
HS_3653	M	25	2	Oligodendroglioma	0	WT	WT			0		0	N	N	Y	N	0	11	Y	N	Y	Y	Neu	0		
HS_3654	M	71	2	Oligodendroglioma	0	WT	WT			0		0,8	N	N	N		1	24	Y	N	Y	Y	Neu	18	C2	
HS_3655	F	65	2	Oligodendroglioma	1	M	WT	WT		0		0,6	N	N	N	N	1	52	Y	N	Y	Y	Neu	17	C5	O3
HS_3656	M	37	2	Oligodendroglioma	0	M	WT			1		0,7	N	N	Y		1	35	Y	N	Y	Y	Mes	17	C5	
HS_3657	F	32	2	Oligodendroglioma	0	M	WT			1		0,95	N	N	Y	N	0	36	Y	N	Y	Y	Cla	17	C4	
HS_3658	M	67	2	Oligodendroglioma	0	WT	WT			0		0,66	N	N	Y		1	25	Y	N	Y	Y	Mes	18	C2	
HS_SBT				Normal															Y	N	Y	N	Mes	17		
HS_SGT				Normal															Y	N		N				
HS_TEM15-4				Normal															Y	N	Y	N	Neu	0		
HS_TEM15-5				Normal															Y	N	Y	N	Neu	0		
HS_TEM15-6				Normal															Y	N		N	Mes	17		
HS_TEM15-7				Normal															Y	N		N	Neu	0		

Supplementary Table 1: Clinical and molecular characteristics the 179 samples obtained through POLA network and used for the study.

A) O1 tumors vs others

P-val rank	Gene Set	GS size	GS size in data	GSA score	GSA pvalue
1	MSIGDB.C2.CGP__CAIRO_PML_TARGETS_BOUND_BY_MYC_UP	23	22	1,154398048	0
9	MSIGDB.C2.CGP__DANG_MYC_TARGETS_UP	143	127	0,778630557	0,001
20	MSIGDB.C2.CGP__MENSSEN_MYC_TARGETS	53	45	0,962255626	0,002
35	MSIGDB.C2.CGP__DANG_REGULATED_BY_MYC_UP	72	67	0,803553674	0,003
40	MSIGDB.C2.CP___PID_MYC_ACTIVPATHWAY	79	75	0,563765617	0,003
58	MSIGDB.C2.CGP__SCHLOSSER_MYC_TARGETS_AND_SERUM_RESPONSE_UP	47	42	1,11079338	0,004
79	ZELLER_MYC_TARGETS	88	74	0,841762915	0,005
99	MSIGDB.C2.CGP__SCHUHMACHER_MYC_TARGETS_UP	80	72	0,984612328	0,006
121	MSIGDB.C2.CGP__COLLER_MYC_TARGETS_UP	25	22	1,090019918	0,007
151	MSIGDB.C3.TFT__V$MYCMAX_B	268	242	0,303364375	0,008
184	MSIGDB.C2.CGP__IRITANI_MAD1_TARGETS_DN	47	42	1,004406513	0,01
210	DOUGHERTY_OPC_MARKERS	291	282	0,506541396	0,011
352	MSIGDB.C2.CGP__BENPORATH_MYC_TARGETS_WITH_EBOX	230	212	0,22215825	0,016
370	MSIGDB.C2.CP___BIOCARTA_CELLCYCLE_PATHWAY	23	23	0,80080082	0,017
489	MSIGDB.C2.CP___BIOCARTA_VEGF_PATHWAY	29	28	0,616193216	0,021
518	MSIGDB.C6.ONCO_MYC_UP.V1_UP	186	172	0,489787022	0,022
599	MSIGDB.C3.TFT__V$MYCMAX_02	268	249	0,17699688	0,025
655	MSIGDB.C2.CGP__SCHLOSSER_MYC_TARGETS_REPRESSED_BY_SERUM	159	156	0,587659985	0,027
656	MSIGDB.C2.CGP__KIM_MYC_AMPLIFICATION_TARGETS_UP	201	185	0,437936039	0,027
688	MSIGDB.C6.ONCO_VEGF_A_UP.V1_DN	193	191	0,415439885	0,028
791	MSIGDB.C2.CGP__RHODES_UNDIFFERENTIATED_CANCER	69	68	1,037258404	0,032
823	MSIGDB.C2.CGP__TOYOTA_TARGETS_OF_MIR34B_AND_MIR34C	463	420	0,435898769	0,033
963	MSIGDB.C2.CGP__SCHLOSSER_MYC_TARGETS_AND_SERUM_RESPONSE_DN	47	41	0,673223517	0,038
1007	MSIGDB.C3.TFT__V$MYCMAX_01	255	240	0,23334513	0,039
1068	MSIGDB.C2.CGP__ODONNELL_TARGETS_OF_MYC_AND_TFRC_DN	45	45	1,018936753	0,042
1099	DIETERICH_GLIOMA_ANGIOGENESIS	95	87	0,913804837	0,043
1195	MSIGDB.C2.CGP__YU_MYC_TARGETS_UP	42	40	1,11307035	0,047
1227	MSIGDB.C2.CGP__MOLENAAR_TARGETS_OF_CCND1_AND_CDK4_DN	58	54	1,001422239	0,048
1269	MSIGDB.C2.CGP__ACOSTA_PROLIFERATION_INDEPENDENT_MYC_TARGETS_UP	84	77	0,342145277	0,049

B) O2 tumors vs others

P-val rank	Gene Set	GS size	GS size in data	GSA score	GSA pvalue
1	MSIGDB.C6.ONCO_CAHOY_NEURONAL	100	96	2,383745508	0
3	GO:0016917=GABA receptor activity	22	21	1,857512018	0
4	GO:0007214=gamma-aminobutyric acid signaling pathway	21	20	1,574603612	0
35	GO:0004890=GABA-A receptor activity	19	18	1,949395581	0,001
46	MSIGDB.C2.CP___REACTOME_GABA_RECEPTOR_ACTIVATION	52	51	0,999101134	0,001
60	MSIGDB.C6.ONCO_CAHOY_ASTROCYTIC	100	98	0,806386292	0,001
113	DOUGHERTY_Neuron	254	247	0,776627777	0,002
138	MSIGDB.C2.CP___REACTOME_GABA_SYNTHESIS_RELEASE_REUPTAKE_AND_DEGRADATION	17	16	2,144759909	0,003
139	MSIGDB.C2.CGP__LEIN_LOCALIZED_TO_DISTAL_AND_PROXIMAL_DENDRITES	17	15	1,780520141	0,003
140	MSIGDB.C2.CGP__LEIN_NEURON_MARKERS	69	66	1,472845054	0,003
143	MSIGDB.C2.CGP__LEIN_LOCALIZED_TO_PROXIMAL_DENDRITES	37	36	1,101427871	0,003
144	GO:0051932=synaptic transmission, GABAergic	28	26	1,038026214	0,003
146	MSIGDB.C5.GO___NEURON_PROJECTION	21	21	1,004926362	0,003
162	MSIGDB.C2.CP___REACTOME_GABA_B_RECEPTOR_ACTIVATION	38	37	0,617725518	0,003
163	GO:0010976=positive regulation of neuron projection development	72	71	0,575794629	0,003
174	GO:0045664=regulation of neuron differentiation	385	368	0,355537857	0,003
230	MSIGDB.C5.GO___NEURON_DEVELOPMENT	61	59	0,483746105	0,005
294	MSIGDB.C5.GO___NEURON_DIFFERENTIATION	76	73	0,478382699	0,007
427	MSIGDB.C5.GO___GENERATION_OF_NEURONS	83	79	0,442067862	0,013
448	GO:0032228=regulation of synaptic transmission, GABAergic	25	23	0,633078639	0,014
962	MSIGDB.C2.CGP__LE_NEURONAL_DIFFERENTIATION_UP	18	17	0,732998262	0,035

C) O3 tumors vs others

P-val rank	Gene Set	GS size	GS size in data	GSA score	GSA pvalue
2	MSIGDB.C2.CGP__RAMALHO_STEMNESS_DN	74	67	1,016166993	0
17	MSIGDB.C2.CGP__LEIN_OLIGODENDROCYTE_MARKERS	74	69	1,938181172	0,001
179	DOUGHERTY_Oligodendrocyte_markers	409	403	1,043910641	0,007
277	GO:0014003=oligodendrocyte development	35	35	0,715154219	0,01
349	MSIGDB.C2.CGP__LEIN_ASTROCYTE_MARKERS	42	40	0,986885835	0,012
514	GO:0048709=oligodendrocyte differentiation	68	68	0,48120876	0,017

Supplementary Table 2: Lists of selected relevant gene sets among all the significantly deregulated gene sets identified with GSA analysis. GSA tests were performed by comparing each of the 1p/19q co-deleted subtypes with the two others (table A for O1, table B for O2 and table C for O3). Pathways are ordered by their p-value rank within the global result list of 19599 gene sets tested. GSA scores reflect the level of up-regulation measured in the given tumour subtype.

	POLA	TCGA	GRAVENDEEL	REMBRANDT
**1p19q co-deleted tumours analysed**	80	131	42	58
**Tumours with available survival data**	80	118	42	37
**Number of death events**	10	13	34	20
**Median survival (months)**	Not reached	134	81,7	80,4

Supplementary Table 3: Comparison of survival data available among patients with 1p19q co-deleted tumours in the 4 cohorts studied (POLA, TCGA, Gravendeel, Rembrandt).

Locus	Target gene	Forward/Reverse	sequence (5'-3')
CICexon1F	CIC	F	AAGACTCGGCAGCATCTCCATGAGGAGGTGCGAGCCC
CICexon1R	CIC	R	GCGATCGTCACTGTTCTCCAACCCCCACCACCGTTACTCC
CICexon2F	CIC	F	AAGACTCGGCAGCATCTCCATCACCTGGCTCCTTTCCA
CICexon2R	CIC	R	GCGATCGTCACTGTTCTCCAAACAGCTGCCGACTCAG
CICexon3F	CIC	F	AAGACTCGGCAGCATCTCCAacctctgtgtccccagAACC
CICexon3R	CIC	R	GCGATCGTCACTGTTCTCCActcagggaactcacGCATC
CICexon4F	CIC	F	AAGACTCGGCAGCATCTCCACAGAGACATGGCCCTCAC
CICexon4R	CIC	R	GCGATCGTCACTGTTCTCCATTCTCCCGCTGCATTAAACA
CICexon5F	CIC	F	AAGACTCGGCAGCATCTCCAgtcctaactgtcccgctctg
CICexon5R	CIC	R	GCGATCGTCACTGTTCTCCAccaagaggcaacagcgtta
CICexon6F	CIC	F	AAGACTCGGCAGCATCTCCAGCCTTCCAGgtaacgctgt
CICexon6R	CIC	R	GCGATCGTCACTGTTCTCCAagagccaaggggctgacta
CICexon7F	CIC	F	AAGACTCGGCAGCATCTCCActgtcatagcgccactctct
CICexon7R	CIC	R	GCGATCGTCACTGTTCTCCActgccctggacaggttcc
CICexon8F	CIC	F	AAGACTCGGCAGCATCTCCAaccctctctgccacctatcc
CICexon8R	CIC	R	GCGATCGTCACTGTTCTCCAggtcctgggcaagattctg
CICexon9F	CIC	F	AAGACTCGGCAGCATCTCCACCTTCCTGGACAGCACTC
CICexon9R	CIC	R	GCGATCGTCACTGTTCTCCAAAACAGACATTCCCATGGCTT
CICexon10F-1	CIC	F	AAGACTCGGCAGCATCTCCATCGGAGTGTCCTGACCTG
CICexon10R-1	CIC	R	GCGATCGTCACTGTTCTCCACTGCCCTGACCAGACTC
CICexon10F-2	CIC	F	AAGACTCGGCAGCATCTCCATCCTCGCCTGCTTCCT
CICexon10R-2	CIC	R	GCGATCGTCACTGTTCTCCACACCAGTGTCTGCAGGAT
CICexon10F-3	CIC	F	AAGACTCGGCAGCATCTCCAACCAGGCCCCTCAGTCAT
CICexon10R-3	CIC	R	GCGATCGTCACTGTTCTCCACATTTGGAGACGCCTCAG
CICexon10F-4	CIC	F	AAGACTCGGCAGCATCTCCACATCGCCTCTAAGCCCTT
CICexon10R-4	CIC	R	GCGATCGTCACTGTTCTCCACTGGGCAATGAACTGGACA
CICexon10F-5	CIC	F	AAGACTCGGCAGCATCTCCAGCCACTGTCACTAACCTACTGG
CICexon10R-5	CIC	R	GCGATCGTCACTGTTCTCCAAAACGGATGCTGGTGGTG
CICexon10F-6	CIC	F	AAGACTCGGCAGCATCTCCAGGAATCACCCAGGTACAGTAC
CICexon10R-6	CIC	R	GCGATCGTCACTGTTCTCCAAAGGGAGCCAAACAGGAC
CICexon11F	CIC	F	AAGACTCGGCAGCATCTCCAtgtttggctcccttgtaacc
CICexon11R	CIC	R	GCGATCGTCACTGTTCTCCAgccaacatccagcaggtaga
CICexon12F	CIC	F	AAGACTCGGCAGCATCTCCAAGGTCTTGGTCTTCCCCT
CICexon12R	CIC	R	GCGATCGTCACTGTTCTCCAATAGGTGATTCTGCCGCAG
CICexon13F	CIC	F	AAGACTCGGCAGCATCTCCATTACCTCACTCCTCCCCATT
CICexon13R	CIC	R	GCGATCGTCACTGTTCTCCAGAAGCAGGAGACCAAGTTAGG

CICexon14F-1	CIC	F	AAGACTCGGCAGCATCTCCAccctaacttggtctcctgctt
CICexon14R-1	CIC	R	GCGATCGTCACTGTTCTCCATGGCCACAGTGTAGACCAG
CICexon14F-2	CIC	F	AAGACTCGGCAGCATCTCCAATTTTACTCTGGCAGCCCTG
CICexon14R-2	CIC	R	GCGATCGTCACTGTTCTCCAAGGTCATGGAACCTGCTAGT
CICexon15F-1	CIC	F	AAGACTCGGCAGCATCTCCATGTCAGATCAACCCAGAGCA
CICexon15R-1	CIC	R	GCGATCGTCACTGTTCTCCACCCCTCAAGCTCAGACTC
CICexon15F-2	CIC	F	AAGACTCGGCAGCATCTCCAAGTCCGAGAGCCAACTGC
CICexon15R-2	CIC	R	GCGATCGTCACTGTTCTCCAtccatgcctgtgtgtctctc
CICexon16F	CIC	F	AAGACTCGGCAGCATCTCCAatctccctgcccatctcc
CICexon16R	CIC	R	GCGATCGTCACTGTTCTCCActtggtggagcgtgttagg
CICexon17F	CIC	F	AAGACTCGGCAGCATCTCCActtggtggagcgtgttagg
CICexon17R	CIC	R	GCGATCGTCACTGTTCTCCActgccctccacctaagga
CICexon18F	CIC	F	AAGACTCGGCAGCATCTCCAAGGAAGAACTCCACGGGTA
CICexon18R	CIC	R	GCGATCGTCACTGTTCTCCACCTTCCAGAGACCCACTC
CICexon19F	CIC	F	AAGACTCGGCAGCATCTCCATCCCCTGTGAAATAGAATGCAG
CICexon19R	CIC	R	GCGATCGTCACTGTTCTCCATACAGATAAGTCCCACCCGA
CICexon20F-1	CIC	F	AAGACTCGGCAGCATCTCCAgctccccaccgtttttctat
CICexon20R-1	CIC	R	GCGATCGTCACTGTTCTCCACTCACCCAGCTCGGACTCT
CICexon20F-2	CIC	F	AAGACTCGGCAGCATCTCCATCCTCTCCCTGTACCGC
CICexon20R-2	CIC	R	GCGATCGTCACTGTTCTCCACCCCACATACTGTCCATGT

Supplementary Table 4: Primers used for amplification of exons 1-20 of *CIC*
